# Multimorbidity in Acute Coronary Syndrome: A Systematic Review and Meta-Analysis

**DOI:** 10.1016/j.jacadv.2025.102006

**Published:** 2025-07-22

**Authors:** Jonathan A. Batty, Tamara del Toro, Daniel J. Drayton, Eleanor Booth, Evrim Anik, Charlotte Sturley, Benjamin C. Brown, Mark T. Kearney, Marlous Hall

**Affiliations:** a Leeds Institute of Cardiovascular and Metabolic Medicine, University of Leeds, Leeds, United Kingdom; b Leeds Institute for Data Analytics, University of Leeds, Leeds, United Kingdom; c Leeds Dental Institute, University of Leeds, Leeds, United Kingdom; d Division of Population Health, Health Services Research and Primary Care, University of Manchester, Manchester, United Kingdom

**Keywords:** acute coronary syndrome, aging, multimorbidity, comorbidity, myocardial infarction

## Abstract

**Background:**

Multimorbidity (the presence of multiple long-term conditions) increases the complexity of management decisions for patients presenting with acute coronary syndrome (ACS).

**Objectives:**

The purpose of this study was to ascertain the prevalence of multimorbidity in ACS and assess its impact on clinical management and outcomes.

**Methods:**

Medline, Web of Science, Embase, and Cochrane were searched to July 2024 for studies that reported: 1) the prevalence of multimorbidity in patients with incident ACS or 2) ACS management and/or clinical outcomes, stratified by multimorbidity status. Random-effects meta-analysis was performed to calculate pooled summary statistics and was supported by narrative synthesis.

**Results:**

Overall, 41 studies were included. Those at low risk of bias (23 studies; *n*_pooled_ = 9,227,657) demonstrated a pooled prevalence of multimorbidity of 46.6% (95% CI: 38.9%-54.2%). Study-level determinants of prevalence included study setting (high-income: 48.5% [40.5%-56.5%] vs low- to middle-income countries: 35.3 [30.5%-40.3%]); *P* = 0.006) and the number of conditions in the per-study definition of multimorbidity (R^2^ = 79.6%; *P* < 0.001). Individual-level determinants of multimorbidity included advanced age, non–ST-segment elevation presentation, previous cardiac procedures, and greater body mass index. Multimorbidity was associated with reduced usage of invasive management and secondary preventative medication. Multimorbidity was associated with short-term mortality (≤30 day; relative risk [RR]_pooled_ 95% CI: 1.43 [95% CI: 1.14-1.78]; *P* < 0.01) and longer-term mortality (>30 day; RR_pooled_: 1.87 [95% CI: 1.51-2.32]; *P* < 0.01). Each additional pre-existing long-term condition was associated with a 16% excess risk of mortality (RR_pooled_: 1.16 [95% CI: 1.06-1.26]; *P* < 0.01).

**Conclusions:**

Multimorbidity is common, associated with reduced use of guideline-directed therapies and adverse clinical outcomes in patients with ACS. (The prevalence of multimorbidity and its impact on clinical outcomes in patients with acute myocardial infarction: a systematic review and meta-analysis; CRD42023447122)

As a result of population aging, an increasing proportion of patients that present with acute coronary syndrome (ACS) have underlying multimorbidity—the coexistence of 2 or more pre-existing long-term conditions (LTCs).^1^^,^^2^ The management of ACS in patients with multimorbidity presents a significant challenge: patients with multimorbidity may be at greater risk of ACS-associated adverse outcomes (including recurrent ischemic events,^3^ mortality,^4^ and worse quality of life^5^) but may also be at greater risk of treatment-related harms (eg, procedural complications^6^ and bleeding^3^). There may exist a risk-treatment paradox, whereby those at the greatest risk (and who may stand to gain the most from interventions) may be less likely to receive them.^3^^,^^4^ Many of the landmark randomized controlled trials of interventions that make up current ACS treatment pathways routinely excluded patients with a significant multimorbidity, limiting evidence-based decision-making in this population.^7^ Although United States,^8^, ^9^, ^10^ European,^11^^,^^12^ and other international clinical guidelines^13^^,^^14^ advocate that multimorbidity should inform clinical decision-making in the context of ACS, the optimal treatment strategy in patients with significant multimorbidity burdens remains unclear. Although instruments that quantify comorbidity burden have been developed for research use,^15^^,^^16^ tools that formalize multimorbidity assessment for the purpose of clinical risk assessment are not established in the context of ACS.

Establishing the current state of the literature regarding the prevalence and impact of multimorbidity in patients presenting with ACS is essential to inform future observational studies and clinical trials that aim to optimize clinical decision-making and management in this population. To date, a number of studies have reported the prevalence and effects of multimorbidity in the context of acute ACS, either directly (based on an a priori definition of multimorbidity) or implicitly (by providing data on comorbidity counts). This review aimed to establish the prevalence of multimorbidity in individuals diagnosed with ACS and the association of multimorbidity with guideline-directed ACS treatment strategies and post-ACS clinical outcomes.

A preliminary search identified a number or related reviews.^17^, ^18^, ^19^, ^20^ However, these either did not have an ACS-specific focus, were undertaken to evaluate the relationship between specific comorbidity measures and outcomes in ACS (such as the Charlson Comorbidity Index), or lacked a systematic methodology. Therefore, this systematic review is the first to comprehensively evaluate the prevalence of multimorbidity and its effect on treatment utilization and clinical outcomes in the context of ACS.

## Methods

This systematic review and meta-analysis was structured in 2 parts, focused on ascertaining: 1) the prevalence of multimorbidity among individuals with ACS; and 2) the association of multimorbidity with treatment strategies, clinical outcomes, and patient-reported outcomes in the post-ACS period.

### Preregistration and reporting

The protocol for this study was prospectively registered (CRD42023447122). It was conducted according to the MOOSE (Meta-analysis Of Observational Studies in Epidemiology guideline) (Supplemental Table 1) and best practices for conducting a systematic review of prevalence.^21^, ^22^, ^23^, ^24^, ^25^ It is reported in line with the Preferred Reporting Items for Systematic Reviews and Meta-Analyses (PRISMA; Supplemental Table 2).^26^^,^^27^ As this study performed secondary analysis of published material, ethical approval was not required.

### Study inclusion and exclusion criteria

The first part of the review included cross-sectional, case-control, or cohort studies that reported the prevalence of multimorbidity in adults (aged ≥18 years) presenting with incident ACS with a definition of multimorbidity that considered ≥2 long-term conditions (or studies which presented disease count data that enabled the calculation of the proportion of individuals with ≥2 long-term conditions). ACS was defined as the presence of ST-segment or non–ST-segment elevation myocardial infarction (MI) or unstable angina. Given the expected heterogeneity of long-term conditions included in studies of this nature and the secondary objective in this review in reporting the long-term conditions included in each per-study definition of multimorbidity, no a priori definition of what constitutes a long-term condition was specified, as outlined in the study protocol.

Studies that restricted their analysis to specific subgroups of ACS (eg, those presenting with cardiogenic shock, multivessel disease, etc), or otherwise demonstrated strong selection bias, were excluded. Studies of ACS patients within particular strata of age (ie, “older patients,” “aged ≥75 years old”) or sex were included. The second part of the review included longitudinal studies that reported the association of multimorbidity status (as defined by individual studies) with one or more clinical or patient-reported post-ACS outcomes. Although it was intended that this would also use a strict definition of ≥2 long-term conditions in the preregistered study protocol, pilot analysis suggested some heterogeneity in the per-study definition of multimorbidity, with some reporting outcome associations with ≥3 long-term conditions. Studies that did not report at least one of the prespecified outcomes (described below) were excluded. No minimum duration of follow-up was required for inclusion.

For both parts of the review, studies were included only if they used contemporaneous ACS diagnostic criteria.^28^, ^29^, ^30^, ^31^ Included studies drew from both primary data sources (eg, prospective cohort studies) and secondary data sources (eg, electronic health record data, national health registries, and administrative databases). The data source used in each study was recorded and considered in the risk of bias assessment. Where multiple studies were identified that described the same population, only the largest of these was included in quantitative synthesis to prevent double counting. Review articles, case reports, and case series were excluded. In order to minimize publication bias, conference proceedings and other “gray literature” were included if they reported the required data, and all eligibility criteria were fulfilled. Studies were eligible if published in the contemporary era of ACS diagnosis and management; 2000–July 2024.^31^ No geographic or language restrictions were applied.

### Study outcomes

The primary outcome was prevalence of pre-existing multimorbidity at the time of ACS. Secondary outcomes included the association between multimorbidity and: 1) treatments received for ACS (including coronary angiography, percutaneous coronary intervention [PCI], and/or coronary artery bypass graft [CABG] surgery, secondary prevention and other pharmacotherapy); 2) post-ACS clinical outcomes (including all-cause mortality, major adverse cardiovascular events [MACE], length of stay, unplanned hospital readmission, and hospital costs); and 3) patient-reported outcome measures (eg, quality of life) in the post-ACS period.

### Data sources and search strategy

Medline (Ovid), Embase (Excerpta Medica), Web of Science (Thomson Reuters), and The Cochrane Database of Systematic Reviews (Cochrane Reviews) were queried from inception. A structured search strategy was developed for Medline (Supplemental Table 3) and adapted to query each database. The final literature search was performed on July 20, 2024. The reference lists of related previous systematic reviews^17^, ^18^, ^19^, ^20^ were also screened. Where eligibility was suggested by screening the title and/or abstract, the full-text article was retrieved and formally assessed against the study inclusion and exclusion criteria. The flow of articles through this process (and stepwise reasons for exclusion) was tracked using Endnote Online (Clarivate). The literature search and data extraction were performed independently by 2 investigators (J.B. and T.dT.), with final arbitration performed in the event of any disagreement (M.H.).

### Risk of bias assessment

The Joanna Briggs Institute Critical Appraisal Checklist for Studies Reporting Prevalence Data (JBI Checklist)^24^ was used to assess the risk of bias in each study that reported the prevalence of multimorbidity in incident ACS.^32^ A minimum sample size requirement was calculated using the method of Naing et al^33^, as recommended by the JBI guidelines.^24^^,^^32^ Assuming a prevalence of multimorbidity of 25% (based on previous work of our group^4^^,^^34^), a two-sided 95% level of confidence, and a desired prevision of 5%, a minimum of *n* = 288 subjects were required to be included in a study for it to be deemed adequately powered. Studies reporting the multimorbidity prevalence in fewer than 288 subjects (the minimum required to produce a reliable estimate), or that failed to satisfy any other domain of the JBI Checklist were excluded. For studies reporting one or more secondary outcomes, the Risk Of Bias In Non-randomized Studies of Exposures checklist was used to assess the risk of bias.^35^ The Risk Of Bias In Non-randomized Studies of Exposures checklist informed to what extent studies should be included in qualitative and narrative analysis, but no absolute bias threshold was set for the exclusion of studies reporting outcome data.

### Data extraction, synthesis, and meta-analysis

Adjusted effect measures were extracted as they account for confounding at a per-study level. Where an OR or HR was reported, these were converted to a relative risk (RR) using the method of Shor et al.^36^ Data presented graphically were extracted using a validated tool.^37^^,^^38^ Quantitative synthesis was performed when ≥5 data points were homogeneously reported for an outcome. Where meta-analysis was not possible, a narrative summary of the findings was made.

Per-study adjusted multimorbidity prevalence estimates were combined to calculate an overall summary prevalence using the Freeman-Tukey (double arcsine square root) transformation under a random effects model, in order to reduce bias from between-study clinical and methodological heterogeneity, and enable greater generalization of study findings.^39^ CIs were calculated using the score statistic method.^40^, ^41^, ^42^ This approach enabled the inclusion of studies that report 0 or 100% multimorbidity, which would be excluded by the standard Wald method. For meta-analysis of dichotomous clinical outcomes, a summary RR and corresponding 95% CI were calculated for each outcome using the DerSimonian and Laird method.^43^ Meta-analyses were performed using the *metaprop* and *meta* packages in Stata (version 17; StataCorp).^44^ The results of both the meta-analyses of multimorbidity prevalence and the impact of multimorbidity on post-ACS clinical outcomes are presented using forest plots. These present per-study multimorbidity estimates and effect sizes, in addition to pooled estimates.

Heterogeneity was quantified using the I^2^, τ^2^, Cochran’s Q, and chi-squared tests. Sources of heterogeneity were explored using prespecified subgroup analysis and meta-regression. Subgroup analyses were performed to evaluate the association of: 1) study age restrictions; 2) ACS subtype; and 3) economic status of study setting with the per-study reported prevalence of multimorbidity. Prespecified meta-regression analyses were performed to identify the association between: 1) age; 2) study period; and 3) the number of long-term conditions included on the per-study reported prevalence of multimorbidity. Missing effects (publication) bias was evaluated using the Begg and Egger tests. Data were presented graphically using forest plots (meta-analysis), funnel plots (missing effect/publication bias), Galbraith plots (heterogeneity), and leave-one-out plots (to assess the possible dominating effect of large studies).

## Results

**Figure 1:**
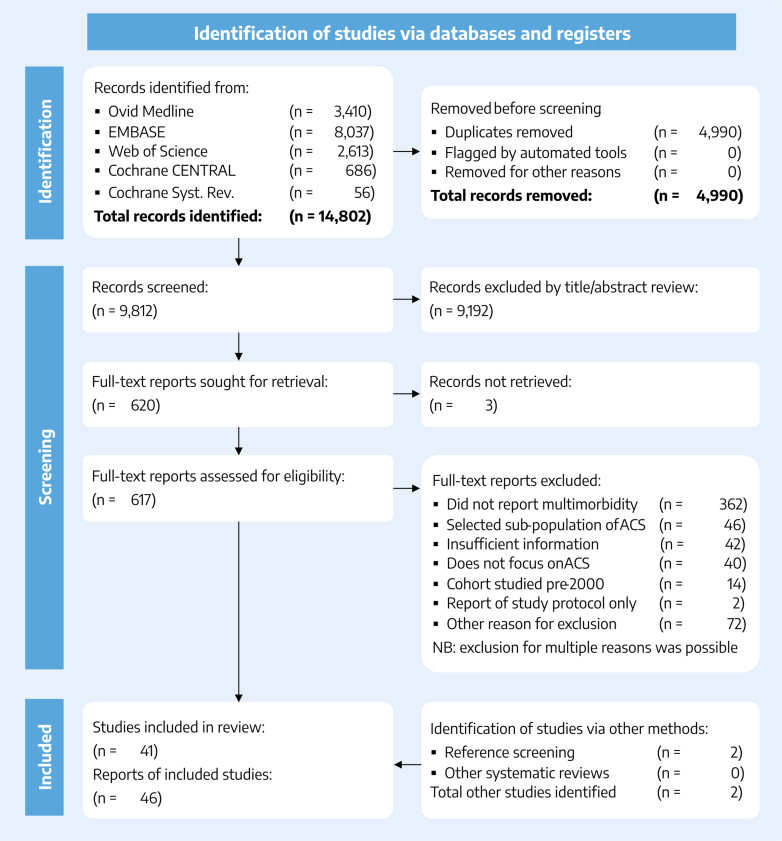
**Summary Flowchart Depicting the Literature Search** The full PRISMA diagram is included in the Supplemental Appendix (Supplemental Figure 1). ACS = acute coronary syndrome; PRISMA = Preferred Reporting Items for Systematic Reviews and Meta-Analyses.

A total of 14,802 articles were identified in the initial literature search (Figure 1, Central Illustration). After the removal of 4,990 duplicates and the exclusion of 9,192 articles on the basis of title and abstract review, 617 full-text reports were retrieved. Of these, 32 met the inclusion criteria. A further 8 studies were identified by searching reference lists: 5 from studies retrieved by the search and 3 from previous related reviews.^17^, ^18^, ^19^, ^20^ Overall, 41 relevant studies were identified, which were described in 46 reports (Table 1).^3^, ^4^, ^5^^,^^34^^,^^39^, ^40^, ^41^, ^42^, ^43^, ^44^, ^45^, ^46^, ^47^, ^48^, ^49^, ^50^, ^51^, ^52^, ^53^, ^54^, ^55^, ^56^, ^57^, ^58^, ^59^, ^60^, ^61^, ^62^, ^63^, ^64^, ^65^, ^66^, ^67^, ^68^, ^69^, ^70^, ^71^, ^72^, ^73^, ^74^, ^75^, ^76^, ^77^, ^78^, ^79^, ^80^ Where multiple studies were identified regarding the same patient population (eg, Hall et al^4^ and Yadegarfar et al^34^) data from only the largest (or most comprehensively reported) study were included in quantitative synthesis. Disagreement over study inclusion for a small number of studies (n = 2) was resolved through discussion (both studies were ultimately included).

**Central Illustration:**
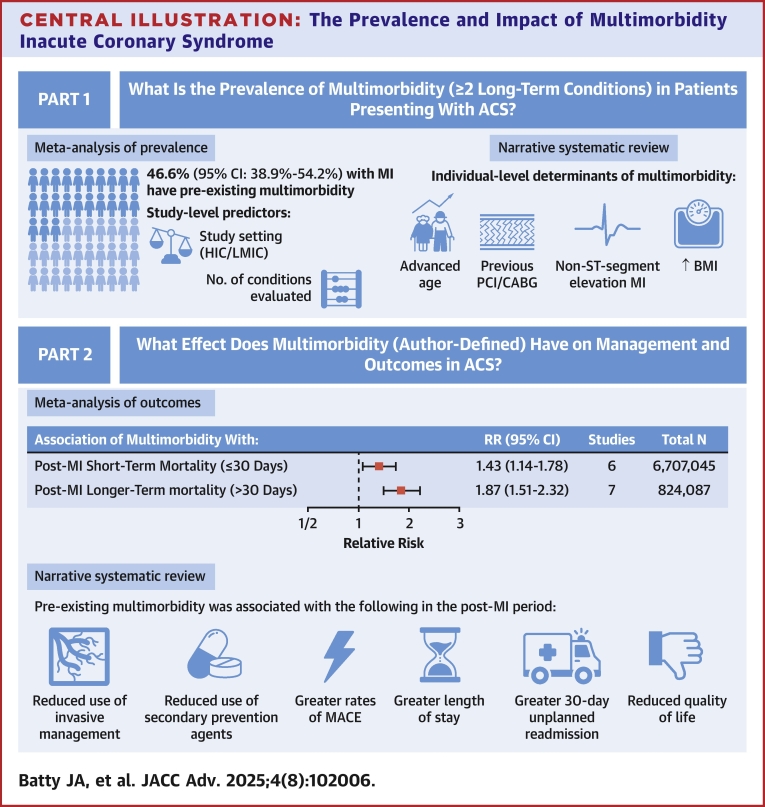
**The Prevalence and Impact of Multimorbidity Inacute Coronary Syndrome** BMI = body mass index; CABG = coronary artery bypass graft; MACE = major adverse cardiovascular events; MI = myocardial infarction; *n* = number; PCI = percutaneous coronary intervention; RR = relative risk; other abbreviations as in Figure 1, Figure 2.

**Table 1:** Summary of Studies and Study Participants

First Author, Year	Study Design; Recruitment Period	Description of Participants		Informed Consent Required	Data Type (Data Source)	Setting, Economic Status	Sample Size (N)	Description of Sample			
		Main Inclusion Criteria	Main Exclusion Criteria					Age (y)	Female (%)	STEMI (%)	NSTE-ACS (%)
Alsawas, 2019^39^	Retrospective cohort study; 1995-2015	Patients admitted to a single hospital with acute MI, identified using ICD-9 codes.	Age <18 y, only first hospitalization included.	ഠ	Secondary, administrative data (Mayo Clinic database).	USA, HIC	15,777	69	35	- - -	- - -
Attar, 2022^40^	Retrospective case-control study; 1995-2013	Patients admitted to hospital with ACS in Denmark, identified using ICD-10 codes.	None reported.	ഠ	Secondary, administrative data (Danish National Patient Registry).	DK, HIC	2,388	67	51	- - -	- - -
Bagai, 2022^41^^,^^42^	Prospective cohort study; 2013-2017	Patients aged ≥65 y, recruited to an international, multicenter registry at 1 – 3 y post-MI.	Life expectancy <1 y; any condition that would limit follow-up.	●	Primary, clinician-collected data (TIGRIS registry).	INT, HIC	5,132	72	27	- - -	- - -
Canivell, 2018^44^	Prospective cohort study; 2009-2014	Patients admitted to one of 4 university hospitals with ACS.	Age <18 y, severe disability, life expectancy <1 y.	●	Primary, clinician-collected data (SPUM-ACS registry).	CH, HIC	5,635	63	21	54	41
Chen, 2015^43^	Retrospective cohort study; 1999-2009	Patients surviving to discharge with acute MI at one of 11 hospitals in Worcester, MA, US.	Age <18 y, death during index hospitalization.	ഠ	Primary, data collected from chart review (Worcester Heart Attack Study).	USA, HIC	3,501	68	42	- - -	- - -
Crane, 2005^45^	Cross-sectional study; Dates not reported	Women with recent MI, aged ≥65 y.	Cognitive impairment or on antidepressants	●	Primary, clinician-collected data (via phone interview).	USA, HIC	84	75	100	- - -	- - -
Dunn, 2009^46^	Prospective cohort study; 2002-2003	Patients admitted to 5 community hospitals with ACS, referred for cardiac rehabilitation.	Age <21 y, non-English speaker, discharge to nonhome setting.	●	Primary, data collected from chart review (HARP study).	USA, HIC	207	59	34	- - -	- - -
Ganasegeran, 2018^47^	Cross-sectional study; 2016	Patients attending follow-up clinic with recent MI (>1 mo)	Age <18 y, cognitive impairment, psychiatric illness, illiteracy.	●	Primary, clinician-collected data.	MY, LMIC	242	55	14	- - -	- - -
Ghushchyan, 2015^48^	Retrospective cohort study; 1998-2009	Patients with an episode of ACS in a national health care survey, identified using ICD-9 codes.	None reported.	●	Secondary, survey interview data (MEPS).	USA, HIC	4,679	67	44	- - -	- - -
Gouda, 2021^49^	Retrospective cohort study; 2010-2016	Patients admitted to hospital with ACS in Alberta, Canada, identified using ICD-10 codes.	Age <18 y; only first episode during study period included.	ഠ	Secondary, administrative data (Alberta Health Care Insurance Plan database).	CA, HIC	31,056	66	31	34	66
Gudnadottir, 2022^3^^,^^50^	Retrospective cohort study; 2006-2013	Patients aged ≥70 y admitted to hospital with ACS.	Only first episode during study period included.	ഠ	Primary, clinical-collected data (SWEDEHEART).	SE, HIC	80,176	80	43	26	74
Gutacker, 2015^51^	Retrospective cohort study; 2008-2009	Patients admitted to publicly funded hospitals in 5 countries, with acute MI, identified using either ICD-9 or 10 codes.	None reported.	ഠ	Secondary, administrative data (ECHO data warehouse).	GB, HIC	69,717	71	36	- - -	- - -
						PT, HIC	12,090	69	35	- - -	- - -
						SI, HIC	3,437	68	36	- - -	- - -
						DK, HIC	7,706	70	35	- - -	- - -
						ES, HIC	51,737	69	30	- - -	- - -
Hall, 2018^4^^,^^34^	Retrospective cohort study; 2003-2013	Patients admitted to hospital in England or Wales with acute MI.	Age <18 y; first MI during study period only.	ഠ	Primary, clinician-collected data (MINAP registry).	GB, HIC	693,388	71	34	40	60
Horne, 2019^52^	Cross-sectional study; Dates not reported	Convenience sample of patients aged ≥65 y, recruited 6 – 8 mo following index MI.	Non-English-speaking, mental or physical disability precluding consent.	●	Primary, written questionnaire data.	USA, HIC	98	76	48	- - -	- - -
Hudzik, 2017^53^^,^^54^	Retrospective cohort study; Dates not reported	Patients with type II DM admitted to hospital with STEMI, undergoing P-PCI.	Patients without type II DM were excluded.	ഠ	Primary, data collected from chart review.	PL, HIC	277	64	41	100	0
Jain, 2022^55^	Retrospective cohort study; 2016-2019	Patients aged ≥66 y and <90 y with Medicare cover admitted to hospital with MI.	Metastatic cancer, Alzheimer disease and related dementia.	ഠ	Secondary, administrative data (Medicare claims data).	USA, HIC	186,012	- - -	- - -	- - -	- - -
Johnman, 2012^56^	Prospective cohort study; 2000-2009	Patients with STEMI that underwent primary or rescue PCI in Scotland, UK.	Only first episode during study period included.	ഠ	Primary, clinician-collected data (Scottish Coronary Revascularisation Register).	GB, HIC	4,354	- - -	26	100	0
Kim, 2023^57^	Prospective cohort study; 2011-2015	Female patients with acute MI admitted to hospital in South Korea	Missing data.	●	Primary, data collected from chart review and via phone.	KR, HIC	3,419	72	100	40	60
King, 2021^58^^,^^59^	Retrospective cohort study; Dates not reported	Patients admitted to a single hospital with acute MI in Hartford, Connecticut, US.	Missing documentation.	ഠ	Primary, data collected from chart review.	USA, HIC	223	64	38	- - -	- - -
McGowan, 2004^60^	Prospective cohort study; Dates not reported	Consecutive patients aged ≥80 y, presenting to 4 urban hospitals with acute MI in the UK.	History of previous MI, psychiatric illness or cognitive impairment.	●	Primary, clinician-collected data.	GB, HIC	305	58	37	- - -	- - -
McManus, 2012^61^	Retrospective cohort study; 1990-2007	Patients surviving to discharge with acute MI at one of 11 hospitals in Worcester, MA, US.	Age <18 y, death during index hospitalization.	ഠ	Primary, data collected from chart review (Worcester Heart Attack Study).	USA, HIC	6,295	70	43	- - -	- - -
Munyombwe, 2021^5^	Prospective cohort study; 2011-2015	Patients admitted to one of 77 hospitals in the UK with a principle diagnosis of acute MI.	Age <18 y, end-stage disease, those not amenable to follow-up.	●	Primary, clinician-collected data (EMMACE-3 and 4 registries).	GB, HIC	8,681	64	25	41	39
Navathe, 2013^62^	Retrospective cohort study; 1997-2004	Patients aged ≥66 and ≤ 90 y with Medicare cover admitted to hospital with acute MI.	LOS <2 days (if discharged alive), HMO enrollment, hospital transfer.	ഠ	Secondary, administrative data (Medicare claims data).	USA, HIC	1,309,554	78	49	- - -	- - -
Nguyen, 2014^63^	Retrospective cohort study; 2010	Patients admitted to a tertiary cardiac hospital with acute MI.	Patients with first MI included only.	ഠ	Secondary, data collected from local EHR system.	VN, LMIC	302	66	33	69	31
Nguyen, 2020^64^	Prospective cohort study; 2018-2019	Consecutive patients aged ≥80 y admitted to 2 cardiac centers with acute MI.	Severe illness, deafness, blindness, dementia, delirium.	●	Primary, clinician-collected data.	VN, LMIC	120	85	50	100	0
Ofori-Asenso, 2019^65^	Retrospective cohort study; 2013-2015	Consecutive patients aged ≥65 y admitted to a hospital in Victoria, Australia for NSTE-ACS.	Nonprimary diagnosis of NSTE-ACS.	ഠ	Secondary, administrative data (Alfred Hospital data).	AU, HIC	1,488	80	38	0	100
Sanchis, 2019^66^	Prospective cohort study; 2002-2012	Patients aged ≥65 y, admitted to a single hospital with NSTE-ACS at one of 2 time periods.	None reported.	●	Primary, clinican-collected data (multiple combined cohort studies)	ES, HIC	920	76	42	0	100
Sanchis, 2021^67^	Prospective cohort study; 2002-2017	Patients aged ≥70 y, admitted to hospital with NSTE-ACS from one of 11 NSTE-ACS registries.	None reported.	●	Primary, clinican-collected data (multiple combined cohort studies)	ES, HIC	7,211	79	38	0	100
Sun, 2020^68^	Retrospective cohort study; 2007-2012	Patients hospitalized with acute MI in Beijing, China.	Age <25 y, nonpermanent residents, LOS ≤1 d, death during admission.	ഠ	Secondary, administrative (Beijing Cardiovascular Disease Surveillance System).	CN, LMIC	64,355	65	30	65	35
Tisminetzky, 2016a^69^	Prospective cohort study; 2011-2013	Patients discharged alive following ACS, from one of 6 centers in MA and GA, US.	Age <21 y, in-hospital death, dementia, imprisonment, pregnancy.	ഠ	Primary, data collected from chart review (TRACE-CORE study).	USA, HIC	2,174	61	33	- - -	- - -
Tisminetzky, 2016b^70^	Retrospective cohort study; 2001-2011	Patients surviving 6 mo postacute MI at one of 3 major hospitals in Worcester, MA, US.	Age <18 y, death during hospitalization or in the subsequent 6 mo.	ഠ	Primary, data collected from chart review (Worcester Heart Attack Study).	US, HIC	4,480	68	41	33	67
Tisminetzky, 2018^71^	Retrospective cohort study; 2001-2011	Patients aged ≥65 y admitted with acute MI at one of 11 hospitals in Worcester, MA, US.	None reported.	ഠ	Primary, data collected from chart review (Worcester Heart Attack Study).	USA, HIC	3,863	79	51	26	74
Tisminetzky, 2019^72^	Retrospective cohort study; 2001-2011	Patients aged ≥65 y admitted with acute MI at one of 3 major hospitals in Worcester, MA, US.	None reported.	ഠ	Primary, data collected from chart review (Worcester Heart Attack Study).	USA, HIC	3,863	79	51	26	74
Tisminetzky, 2021^73^	Retrospective cohort study; 2003-2015	Patients surviving to discharge with acute MI at one of 3 major hospitals in Worcester, MA, US.	Age <18 y, death during hospitalization. Patients with first MI only.	ഠ	Primary, data collected from chart review (Worcester Heart Attack Study).	USA, HIC	3,116	67	42	36	64
Turner, 2020^74^	Prospective cohort study; 2008-2013	Patients admitted to one of 16 centers across the UK with NSTE-ACS.	Life expectancy <1 y, no fixed address or GP, inability to consent.	●	Primary, clinician-collected data (PhACS study).	GB, HIC	1,456	65	27	0	100
Worrall-Carter, 2016a^75^	Retrospective cohort study; 2007-2009	Patients admitted to hospital in Victoria, Australia NSTE-ACS, identified using ICD-10 codes.	Patients with UA without a high-risk comorbidity (HF, arrhythmia, CKD, DM).	ഠ	Secondary, administrative data (Victorian Admitted Episodes Dataset).	AU, HIC	16,771	- - -	38	0	100
Worrall-Carter, 2016b^76^	Retrospective cohort study; 2007-2009	Patients admitted to hospital in Victoria, Australia with ACS, identified using ICD-10 codes.	Patients with first MI included only.	ഠ	Secondary, administrative data (Victorian Admitted Episodes Dataset).	AU, HIC	28,985	- - -	36	18	82
Yan, 2022^77^	Prospective cohort study; 2003-2014	Patients aged ≥65 y admitted to hospital with ACS, recruited to an international, multicenter registry at one of 15 centers.	None reported.	●	Primary, clinican-collected data (BleeMACS registry)	INT, HIC	7,120	- - -	- - -	- - -	- - -
Yang, 2011^78^	Retrospective cohort study; 1993-2007	Patients admitted to a single hospital in Beijing, China with acute MI, identified using ICD-9.	Nonprimary diagnosis of ACS.	ഠ	Secondary, data collected from local EHR system.	CN, LMIC	5,161	64	20	- - -	- - -
Zhang, 2020^79^	Retrospective cohort study; 2004-2014	Patients discharged from a hospital included in the US NIS with a primary diagnosis of ACS.	Age <18 y, nonprimary diagnosis of ACS.	ഠ	Secondary, administrative data (HCUP Nationwide Inpatient Sample).	USA, HIC	6,613,623	67	40	36	64
Zykov, 2022^80^	Retrospective cohort study; 2016 - 2017	Consecutive patients admitted to a single hospital in Sochi, Russia with ACS	None reported.	ഠ	Primary, data collected from chart review.	RU, HIC	2,305	67	40	30	70

Multiple references are given where multiple related publications have resulted from the same data.

ACS = acute coronary syndrome; AU = Australia; BleeMACS = Bleeding complications in a Multicenter registry of patients discharged after an Acute Coronary Syndrome; CA = Canada; CN = China; DK = Denmark; DM = diabetes mellitus; EHR = electronic health records; ES = Spain; GA = Georgia; GB = Great Britain; GP = general practitioner; HARP = Heart After Hospital Recovery Planner; HCUP = Healthcare Cost and Utilization Project; HIC = high-income country; HMO = health maintenance organization; ICD = International Classification of Diseases; INT = international cohort; LMIC = low- to middle-income country; LOS = length of stay; MA = Massachusetts; MEPS = Medical Expenditure Panel Survey; MI = myocardial infarction; MINAP = Myocardial Ischaemia National Audit Project; MY = Malaysia; n = number; NIS = Nationwide Inpatient Sample; NSTE-ACS = non–ST-segment elevation acute coronary syndrome; PL = Poland; PT = Portugal; RU = Russia; SE = Sweden; SI = Slovenia; SPUM-ACS = Special Program University Medicine-Acute Coronary Syndromes study; STEMI = ST-segment elevation myocardial infarction; SWEDEHEART = Swedish Web-system for Enhancement and Development of Evidence-based care in Heart disease Evaluated According to Recommended Therapies; TIGRIS = long-Term rIsk, clinical manaGement, and healthcare Resource utilisation of stable coronary artery dISease; USA = United States of America; VN = Vietnam. Footnotes: data that were not reported (or are otherwise missing) are represented by dashes in the relevant table cells (-- -). Key: ഠ – consent not required; ● – consent was required.

### Study characteristics

The characteristics of the included studies are presented in Table 1. These were published between 2004 and 2022. Of the included studies, 27 reported their findings based on primary data collection^3^, ^4^, ^5^^,^^34^^,^^41^, ^42^, ^43^, ^44^, ^45^, ^46^, ^47^^,^^50^^,^^52^, ^53^, ^54^^,^^56^, ^57^, ^58^, ^59^, ^60^, ^61^^,^^64^^,^^66^^,^^67^^,^^69^, ^70^, ^71^, ^72^, ^73^, ^74^^,^^77^^,^^80^ and 14 reported secondary analyses of routinely collected data.^39^^,^^40^^,^^48^^,^^49^^,^^51^^,^^55^^,^^62^^,^^63^^,^^65^^,^^68^^,^^75^^,^^76^^,^^78^^,^^79^ Secondary data sources included administrative data,^39^^,^^40^^,^^49^^,^^51^^,^^55^^,^^62^^,^^65^^,^^68^^,^^75^^,^^76^^,^^79^ electronic health records,^63^^,^^78^ and national survey data.^48^ There were 36 cohort studies (12 prospective^5^^,^^41^^,^^42^^,^^44^^,^^46^^,^^56^^,^^57^^,^^60^^,^^64^^,^^66^^,^^67^^,^^69^^,^^74^^,^^77^ and 24 retrospective,^3^^,^^4^^,^^34^^,^^39^^,^^43^^,^^48^, ^49^, ^50^, ^51^^,^^53^, ^54^, ^55^^,^^58^^,^^59^^,^^61^, ^62^, ^63^^,^^65^^,^^68^^,^^70^, ^71^, ^72^, ^73^^,^^75^^,^^76^^,^^78^, ^79^, ^80^) 3 cross-sectional studies,^45^^,^^47^^,^^52^ and one case-control study.^40^ Sixteen studies were performed in the United States,^39^^,^^43^^,^^45^^,^^46^^,^^48^^,^^52^^,^^55^^,^^58^^,^^59^^,^^61^^,^^62^^,^^69^, ^70^, ^71^, ^72^, ^73^^,^^79^ 5 in the United Kingdom,^4^^,^^5^^,^^34^^,^^56^^,^^60^^,^^74^ 3 in Australia^65^^,^^75^^,^^76^ and international cohorts,^41^^,^^42^^,^^51^^,^^77^ 2 in China,^68^^,^^78^ Spain,^66^^,^^67^ and Vietnam,^63^^,^^64^ and one in each of Canada,^49^ Denmark,^40^ Malaysia,^47^ Poland,^53^^,^^54^ Russia,^80^ Sweden,^3^^,^^50^ South Korea,^57^ and Switzerland.^44^ All but 3 studies^68^^,^^77^^,^^80^ were reported in English. Translations of the non-English studies were successfully obtained.

Fourteen studies restricted their analysis to older adults (age ≥65 years,^41^^,^^42^^,^^45^^,^^52^^,^^65^^,^^66^^,^^71^^,^^72^^,^^77^ 66-90 years,^55^^,^^62^ ≥70 years,^3^^,^^50^^,^^67^ and ≥80 years^60^^,^^64^). Twenty-three studies included patients presenting with acute MI,^4^^,^^5^^,^^34^^,^^39^^,^^41^, ^42^, ^43^^,^^45^^,^^47^^,^^51^^,^^52^^,^^55^^,^^57^, ^58^, ^59^, ^60^, ^61^, ^62^, ^63^, ^64^^,^^68^^,^^70^, ^71^, ^72^, ^73^^,^^78^ 11 included patients with a diagnosis of ACS,^3^^,^^40^^,^^44^^,^^46^^,^^48^, ^49^, ^50^^,^^69^^,^^76^^,^^77^^,^^79^^,^^80^ 5 included patients with non–ST-segment elevation (NSTE)-ACS only^65^, ^66^, ^67^^,^^74^^,^^75^ and 2 included patients with ST-segment elevation MI (STEMI) only.^53^^,^^54^^,^^56^

### Participant characteristics

Study sample sizes ranged from 84^45^ to 6,613,623^79^ subjects. The mean age ranged from 55^47^ to 85^64^ years. Five studies did not report data on age.^55^^,^^56^^,^^75^, ^76^, ^77^ Most subjects were male (49% to 86%).^40^^,^^47^^,^^71^^,^^72^ One study was restricted to female patients.^57^ Two studies did not report the proportion of patients included by sex.^55^^,^^77^

### Assessment of multimorbidity

The included studies differed in how many long-term conditions they included in their ascertainment of multimorbidity (Table 2), ranging from 3^47^ to 53^55^ (median = 10). The long-term conditions evaluated by each study also differed markedly. Disease states that were most commonly included were diabetes mellitus (39 studies), chronic kidney disease (33 studies), and hypertension (33 studies). All 3 of these were reported in 26 studies. Other long-term conditions were included with greater heterogeneity. Several studies were limited to the conditions included in pre-existing comorbidity indices (such as the Charlson Comorbidity Index).

**Table 2:** The Definition of Multimorbidity Used by Included Studies

First Author, Year	Reporting of Multimorbidity	Reported Prevalence of Multimorbidity in MI (%)	No. of Disease States Included in Definition	Diabetes Mellitus	CKD and/or ESRF	Hypertension	Cerebrovascular Disease	Chronic Pulmonary Disease	Heart Failure	Peripheral Vascular Disease	Anemia	Coronary Artery Disease^a^	Arrhythmia (Including AF)	Cancer^b^	Depression	Systemic GI/Liver Disease	Dyslipidemia	Obesity	Valvular Heart Disease	Dementia	Other Diseases Included in Definition
Jain, 2022^55^	Proportion (% of participants) with <2 and ≥2 chronic diseases.	86.6	53	●	●	●	●	●	●	●	●	●	●	●	●	●	●	●	ഠ	ഠ	See corresponding footnote for description of all included disease states^d^
Gutacker^c^, 2015^51^	Proportion (% of participants) with 0, 1, 2-3, and ≥4 Elixhauser comorbidities.	49.7	31	●	●	●	ഠ	●	●	ഠ	●	ഠ	●	●	●	●	ഠ	●	●	ഠ	PHT, paralysis, hypothyroidism, PUD, HIV/AIDS, lymphoma, metastasis, RA/CTD, coagulopathy, weight loss, fluid/electrolyte disorders, alcohol/drug abuse, psychoses.
Worrall-Carter, 2016a^75^	Proportion (% of participants) with 0, 1, 2, and ≥3 Elixhauser comorbidities.	61.4	30	●	●	●	ഠ	●	●	●	●	●	ഠ	●	●	●	ഠ	●	●	ഠ	PHT, paralysis, hypothyroidism, PUD, HIV/AIDS, lymphoma, metastasis, RA/CTD, coagulopathy, weight loss, fluid/electrolyte disorders, alcohol/drug abuse, psychoses, other neurological disorders.
Worrall-Carter, 2016b^76^	Proportion (% of participants) with 0, 1, 2, and ≥3 Elixhauser comorbidities.	48.4	30	●	●	●	ഠ	●	●	●	●	●	ഠ	●	●	●	ഠ	●	●	ഠ	PHT, paralysis, hypothyroidism, PUD, HIV/AIDS, lymphoma, metastasis, RA/CTD, coagulopathy, weight loss, fluid/electrolyte disorders, alcohol/drug abuse, psychoses, other neurological disorders.
Zhang, 2020^79^	Proportion (% of participants) with 0, 1, 2, 3, 4, and ≥5 Elixhauser comorbidities.	66.1	29	●	●	●	ഠ	●	●	●	●	ഠ	ഠ	●	●	●	ഠ	●	●	ഠ	PHT, paralysis, hypothyroidism, PUD, HIV/AIDS, lymphoma, metastasis, RA/CTD, coagulopathy, weight loss, fluid/electrolyte disorders, alcohol/drug abuse, psychoses, other neurological disorders.
Navathe, 2013^62^	Proportion (count and % of participants) with 0, 1, 2, 3, and ≥4 Elixhauser comorbidities.	59.2	27	●	●	●	ഠ	●	●	ഠ	●	ഠ	●	●	●	●	ഠ	●	●	ഠ	PHT, paralysis, hypothyroidism, PUD, HIV/AIDS, lymphoma, metastasis, RA/CTD, weight loss, alcohol/drug abuse, psychoses, neurodegenerative disorders.
Horne, 2019^52^	Proportion (count of participants) with 2-11 comorbidities (including MI; subtracted)	96.9	23	●	●	●	●	●	●	●	ഠ	●	ഠ	●	ഠ	●	●	ഠ	ഠ	ഠ	Hemiplegia, asthma/rheumatism, migraine, asthma/rhinitis, RA, Parkinson disease, epilepsy, acne, ulcers, glaucoma, gout, TB.
Alsawas, 2019^39^	Proportion (% of participants) with ≥2 existing chronic conditions.	48.6	20	●	●	●	●	●	●	ഠ	ഠ	●	●	●	●	●	●	ഠ	ഠ	●	Arthritis, substance abuse, osteoporosis, schizophrenia, autism, asthma, HIV/AIDS.
Gutacker, 2015^51^	Proportion (% of participants) with 0, 1, 2-3, and ≥4 Charlson comorbidities.	20.9	17	●	●	ഠ	●	●	●	●	ഠ	●	ഠ	●	ഠ	●	ഠ	ഠ	ഠ	●	Rheumatologic disease, PUD, hemiplegia or paraplegia, metastasis, HIV/AIDS.
Dunn, 2009^46^	Proportion (count and % of participants) with 0, 1, 2, 3, and ≥4 Charlson conditions.	46.4	17	●	●	ഠ	●	●	●	●	ഠ	●	ഠ	●	ഠ	●	ഠ	ഠ	ഠ	●	CTD, PUD, hemiplegia, leukemia, lymphoma, metastasis, HIV/AIDS.
Tisminetzky, 2016a^69^	Proportion (count of participants) with 0 – 1, 2-3, and ≥4 comorbidities.	78.3	16	●	●	●	●	●	●	●	●	ഠ	●	●	●	●	●	ഠ	●	ഠ	Arthritis, anxiety.
Turner, 2020^74^	Proportion (% of participants) with 0, 1, 2, and ≥3 pre-existing diseases.	49.9	15	●	●	●	●	●	ഠ	●	ഠ	●	ഠ	●	ഠ	●	ഠ	ഠ	ഠ	●	SAH, PUD, OA, CTD (RA, PMR), chronic neurological conditions (epilepsy, MND, Parkinson disease).
Hudzik, 2017^53^^,^^54^	Proportion (% of participants) with 0-6 comorbidities (all patients had DM).	79.1	14	●	●	●	●	●	●	●	●	ഠ	●	ഠ	●	ഠ	●	ഠ	ഠ	ഠ	Asthma, PUD, or GI bleed, hypothyroidism/hyperthyroidism or goiter, depression, CTD.
Canivell, 2018^44^	Proportion (count of participants) with ≥2 pre-existing cardiovascular comorbidities, noncardiovascular comorbidities (or both).	35.0	13	●	●	●	●	●	●	●	ഠ	●	ഠ	●	ഠ	●	ഠ	ഠ	ഠ	ഠ	Familial hypercholesterolemia, GI bleed, inflammatory disease (SLE, PMR, RA, polymyositis, mixed CTDs, or psoriasis).
Ofori-Asenso, 2019^65^	Proportion (count of participants) with 0, 1, and ≥2 noncardiovascular comorbidities.	20.9	13	●	●	ഠ	ഠ	●	ഠ	ഠ	●	ഠ	ഠ	●	●	●	ഠ	●	ഠ	●	CTD, PUD, HIV/AIDS, psychoses.
Attar, 2022^40^	Proportion (% of participants) with 0, 1, and ≥2 conditions at baseline.	39.0	13	●	ഠ	●	●	●	●	●	●	ഠ	●	ഠ	ഠ	ഠ	●	●	●	ഠ	Cardiomyopathy, sick sinus syndrome.
Bagai, 2022^41^^,^^42^	Proportion (count and % of participants) with 0-9 comorbid conditions.	36.5	12	●	●	ഠ	●	●	●	●	●	●	●	●	●	ഠ	ഠ	ഠ	ഠ	ഠ	None.
Gudnadottir, 2022^3^^,^^50^	Proportion (% of participants) with <2 and ≥2 chronic diseases.	67.5	11	●	●	●	●	●	●	●	●	●	●	●	ഠ	ഠ	ഠ	ഠ	ഠ	ഠ	None.
Tisminetzky, 2019^72^	Proportion of participants in group 3 (≥3 cardiac-related comorbidities) and 4 (≥3 cardiac and ≥1 noncardiac comorbidity)	51.1	11	●	●	●	●	●	●	ഠ	●	●	●	ഠ	●	ഠ	●	ഠ	ഠ	ഠ	None.
Tisminetzky, 2021^73^	Proportion (% of participants) with ≤1, 2, and ≥3 pre-existing comorbidities.	51.3	11	●	●	●	●	●	●	●	●	ഠ	●	ഠ	●	ഠ	●	ഠ	ഠ	ഠ	None.
Yan, 2022^77^	Proportion (count of participants) with 1, 2, and ≥3 morbidities.	67.4	10	●	●	●	●	ഠ	●	●	ഠ	●	ഠ	●	ഠ	ഠ	●	ഠ	ഠ	ഠ	Previous bleeding.
Yang, 2011^78^	Proportion (count of participants) with 0, 1, 2, and ≥3 pre-existing comorbidities.	37.5	9	●	●	●	●	●	ഠ	ഠ	●	●	●	ഠ	ഠ	ഠ	ഠ	ഠ	●	ഠ	None.
Tisminetzky, 2016b^70^	Proportion (count and % of participants) with 0, 1, 2, 3, and ≥4 morbidities.	54.7	9	●	●	●	●	●	●	●	●	ഠ	●	ഠ	ഠ	ഠ	ഠ	ഠ	ഠ	ഠ	None.
Zykov, 2022^80^	Proportion (count of participants) with 0-1, 2-3 and ≥4 diseases.	78.9	9	●	●	●	●	ഠ	ഠ	●	●	ഠ	●	ഠ	ഠ	ഠ	ഠ	●	ഠ	ഠ	Thrombocytopenia.
Sun, 2020^68^	Proportion (% of participants) with 1, 2, 3, 4, and ≥5 comorbidities.	41.6	8	●	●	●	●	ഠ	●	ഠ	ഠ	ഠ	●	ഠ	ഠ	ഠ	●	ഠ	ഠ	ഠ	History of pneumonia.
Hall, 2018^4^^,^^34^	Proportion (count and % of participants) with ≥1, 1, 2, and ≥3 chronic conditions.	25.2	7	●	●	●	●	●	●	●	ഠ	ഠ	ഠ	ഠ	ഠ	ഠ	ഠ	ഠ	ഠ	ഠ	None.
Munyombwe, 2021^5^	Proportion (count and % of participants) with 0, 1, 2, 3, 4, and ≥5 comorbidities.	22.6	7	●	●	●	●	●	●	●	ഠ	ഠ	ഠ	ഠ	ഠ	ഠ	ഠ	ഠ	ഠ	ഠ	None.
Nguyen, 2014^63^	Proportion (% of participants) with 0, 1, and ≥2 prior cardiovascular comorbidities.	23.8	7	●	ഠ	●	●	ഠ	●	ഠ	ഠ	●	●	ഠ	ഠ	ഠ	●	ഠ	ഠ	ഠ	None.
Nguyen, 2020^64^	Proportion (% of participants) with <2 and ≥2 chronic diseases.	72.5	6	●	●	●	●	ഠ	●	ഠ	ഠ	●	ഠ	ഠ	ഠ	ഠ	ഠ	ഠ	ഠ	ഠ	None.
McGowan, 2004^60^	Proportion (count and % of participants) with 0, 1, 2, 3, and 4 comorbidities.	41.3	6	ഠ	ഠ	●	ഠ	●	ഠ	ഠ	ഠ	●	ഠ	ഠ	ഠ	ഠ	●	ഠ	ഠ	ഠ	Rheumatological disorders, neurological disorders.
Tisminetzky, 2018^71^	Proportion (% of participants) with ≥2 pre-existing noncardiovascular comorbidities.	33.5	6	●	●	ഠ	ഠ	●	ഠ	ഠ	●	ഠ	ഠ	ഠ	●	ഠ	ഠ	ഠ	ഠ	●	None.
Sanchis, 2019^66^	Proportion (count of participants) with 0, 1, 2, 3, 4, 5, and 6 comorbidities.	50.2	6	●	●	ഠ	●	●	ഠ	●	●	ഠ	ഠ	ഠ	ഠ	ഠ	ഠ	ഠ	ഠ	ഠ	None
Sanchis, 2021^67^	Proportion (count and % of participants) with 0, 1, 2, 3, 4, and 5-6 comorbidities.	40.3	6	●	●	ഠ	●	●	ഠ	●	●	ഠ	ഠ	ഠ	ഠ	ഠ	ഠ	ഠ	ഠ	ഠ	None
Gouda, 2021^49^	Proportion (count and % of participants) with 1, 2, and ≥3 comorbidities.	31.3	6	●	●	●	●	ഠ	●	●	ഠ	ഠ	ഠ	ഠ	ഠ	ഠ	ഠ	ഠ	ഠ	ഠ	None.
Chen, 2015^43^	Proportion (% of participants) with ≥2 pre-existing comorbidities.	36.6	5	●	ഠ	●	●	ഠ	●	ഠ	ഠ	●	ഠ	ഠ	ഠ	ഠ	ഠ	ഠ	ഠ	ഠ	None.
Johnman, 2012^56^	Proportion (count and % of participants) with ≥2 comorbidities.	34.5	5	●	●	●	●	ഠ	ഠ	●	ഠ	ഠ	ഠ	ഠ	ഠ	ഠ	ഠ	ഠ	ഠ	ഠ	None.
Kim, 2023^57^	Proportion (count and % of participants) with ≥0, 1, 2, 3, 4, and 5 comorbid diseases.	42.0	5	●	ഠ	●	●	ഠ	ഠ	ഠ	●	ഠ	ഠ	ഠ	ഠ	ഠ	●	ഠ	ഠ	ഠ	None.
King, 2021^58^^,^^59^	Proportion (count and % of participants) with 0, 2, 3, and 4-5 comorbidities.	66.4	5	●	●	●	ഠ	ഠ	ഠ	ഠ	ഠ	ഠ	●	ഠ	ഠ	ഠ	●	ഠ	ഠ	ഠ	None.
McManus, 2012^61^	Proportion (count and % of participants) with ≥1, 1, 2, 3, and ≥ 4 comorbidities.	45.6	5	●	ഠ	●	●	ഠ	●	ഠ	ഠ	ഠ	●	ഠ	ഠ	ഠ	ഠ	ഠ	ഠ	ഠ	None.
Ganasegeran, 2018^47^	Proportion (count and % of participants) with <2 and ≥ 2 comorbidities.	63.6	3	●	ഠ	●	ഠ	ഠ	ഠ	ഠ	ഠ	ഠ	ഠ	ഠ	ഠ	ഠ	●	ഠ	ഠ	ഠ	None.
Ghushchyan, 2015^48^	Proportion (% of participants) with 0, 1, 2, 3, 4, and ≥ 5 chronic conditions.	88.2	6																		Details not reported.
Crane, 2005^45^	Count of self-reported comorbidities in the “Demographic Health Status Tool.”	83.3	NR																		Details not reported.
Total times disease was included in per-study definition				38	33	32	29	28	28	24	20	18	18	17	14	14	14	9	8	6	(of 39 reported per-study definitions)

This table is arranged in descending order by number of diseases evaluated. Key: ● condition included in definition; ഠ condition not included in definition;  not reported.

AF = atrial fibrillation; CKD = chronic kidney disease; CTD = connective tissue disease; DM = diabetes mellitus; ESRF = end-stage renal failure; GI = gastrointestinal; HIV/AIDS = human immunodeficiency virus/acquired immune deficiency syndrome; MND = motor neurone disease; NR = not reported; OA = osteoarthritis; PHT = pulmonary hypertension; PMR = polymyalgia rheumatica; PUD = peptic ulcer disease; RA = rheumatoid arthritis; SAH = subarachnoid hemorrhage; SLE = systemic lupus erythematosus; TB = tuberculosis; other abbreviation as in Table 1.

a CAD includes previous MI, angina pectoris, previous PCI, or CABG.

b cancer refers to any solid organ malignancy, leukemia or lymphoma, without metastasis.

c Gutacker et al (2015)^51^ report 2 definitions, based on the implementation of both the Elixhauser and Charlson Indices. Both are reported separately in this table.

d Other endocrine/metabolic disorders, history of acute heart/respiratory failure, acute renal failure, thrombocytopenia and other hematological disorders, other depressive disorders, RA and inflammatory CTDs, asthma, major depressive/bipolar/paranoid disorders, chronic nonpressure skin ulcers, protein-calorie malnutrition, complications of implants or grafts, sepsis or septic shock, substance use disorder, pneumonias, home oxygen use, other trauma, disorders of immunity, liver diseases, severe cancers, seizure disorders and convulsions, other neurological diseases, home hospital bed or wheelchair use, pressure ulcer of skin, amputation and complications, IBD, Parkinson and Huntington disease, spinal cord and paralytic disorders, major organ transplant, severe hematological disorders, schizophrenia and psychosis, artificial openings for feeding or elimination, head trauma, opportunistic infections, chronic pancreatitis, HIV/AIDS, respirator dependence and tracheostomy.

### Prevalence of multimorbidity at ACS presentation

**Figure 2:**
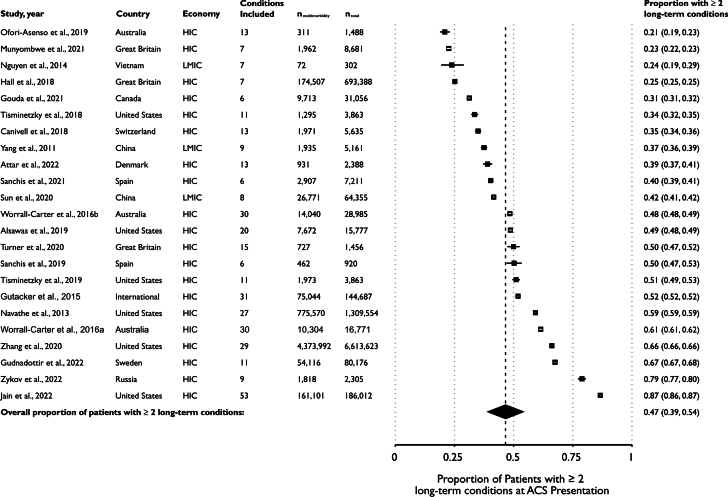
**Prevalence of ≥2 Pre-Existing Long-Term Conditions at ACS by Study** A random effects model was used, implementing the Freeman-Tukey (double arcsine square root) transformation. CIs were calculated using the score statistic method and are shown in black if outside the effect size marker, and white within the marker. Note: CIs are very small for many individual studies owing to their large sample sizes. These may not be visible behind the study marker. n_multimorbidity_ = number of patients with ≥2 long-term conditions at ACS diagnosis in study; HIC = high-income country; LMIC = low- to middle-income country; n_total_ = total number of patients with MI in study, other abbreviation as in Figure 1.

The overall prevalence of multimorbidity (≥2 long-term conditions) in those presenting with acute ACS was 46.6% (95% CI: 38.9%-54.2%) (Figure 2), with high between-study heterogeneity (I^2^ = 99%; *P* < 0.001). This was calculated from 23 studies that reported the prevalence of pre-existing multimorbidity with minimal bias (Supplemental Table 4).^3^, ^4^, ^5^^,^^34^^,^^39^^,^^40^^,^^44^^,^^49^, ^50^, ^51^^,^^55^^,^^62^^,^^63^^,^^65^, ^66^, ^67^, ^68^^,^^71^^,^^72^^,^^74^, ^75^, ^76^^,^^78^, ^79^, ^80^ Meta-analysis stratified by the presence of study age restrictions did not demonstrate a significant difference in multimorbidity prevalence, which was 51.5% (38.4-64.5) for studies that included older patients only (8 studies^3^^,^^50^^,^^55^^,^^62^^,^^65^, ^66^, ^67^^,^^71^^,^^72^ vs 46.6% (39.2-54.0) for studies of unselected patients (*P*_interaction_ = 0.423). Similarly, there was no significant difference for studies that recruited NSTE-ACS only vs all ACS (*P*_interaction_ = 0.719). However, studies conducted in high-income countries reported a higher prevalence than those from low- and middle-income countries: high-income country 48.5% (40.5%-56.5%) vs low- and middle-income country 35.3 (30.5%-40.3%) (*P*_interaction_ = 0.006).

**Figure 3:**
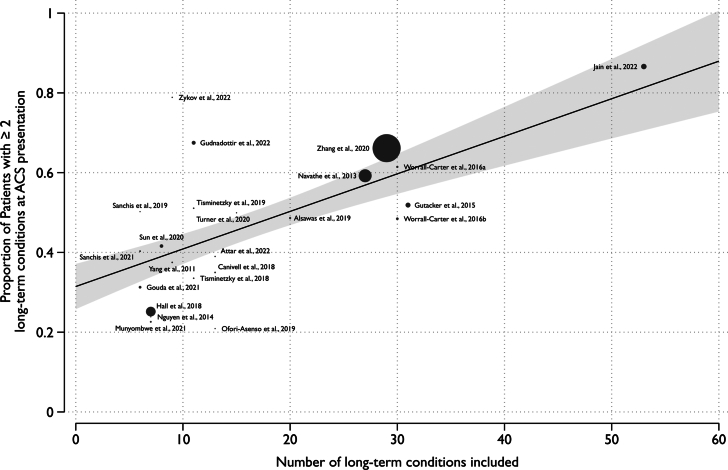
**Association Between Long-Term Conditions Studied and Reported Prevalence of Multimorbidity** Trend line calculated using DerSimonian and Laird (inverse variance) weighting method. Summary of meta-regression: β = 0.009 (95% CI: 0.007-0.012); *P* < 0.001; R^2^ = 79.6%. Abbreviation as in Figure 1.

Random effects meta-regression demonstrated no association between the age of study participants (β = 0.004 [95% CI: −0.014 to 0.021]; *P* = 0.700; R^2^ = 0%) or the median year of the study period (β = 0.004 [95% CI: −0.014 to 0.021]; *P* = 0.678; R^2^ = 0%) with the per-study multimorbidity prevalence (Supplemental Figure 3). However, there was a significant association between the number of comorbidities that study included in its definition of multimorbidity and multimorbidity prevalence (β = 0.009 [95% CI: −0.007 to 0.012]; *P* < 0.001; R^2^ = 79.6%) (Figure 3).

Studies that reported individual-level determinants of multimorbidity^3^^,^^39^^,^^44^^,^^50^^,^^63^^,^^65^ suggested that pre-existing multimorbidity at ACS presentation was associated with: 1) advanced age; 2) NSTE-ACS presentation; 3) previous cardiovascular procedures (including prior PCI and CABG); and 4) higher body mass index (BMI). Among 5,635 patients admitted with ACS in Switzerland, those with multimorbidity were older (65.8 vs 61.8 years; *P* < 0.001), more likely to be female (22.4 vs 19.6%; *P* = 0.01) and less likely to have received education to high school level or beyond (21.7 vs 28.2%; *P* < 0.001). No difference was noted by ethnicity (93.6 non-Caucasian vs 94.5% Caucasian; *P* > 0.05), smoking status (29.9 vs 31.3% never smokers; *P* > 0.05), or alcohol consumption (>14 U/week; 12.2 vs 12.8%; *P* > 0.05).^44^ In 15,777 patients hospitalized with MI in the United States, multimorbidity was more common in women compared to men (52.2 vs 46.7%).^39^ In an analysis of SWEDEHEART (Swedish Web-system for Enhancement and Development of Evidence-based care in Heart disease Evaluated According to Recommended Therapies) stratified by ACS subtype (STEMI, n = 20,540; NSTE-ACS, n = 59,636), those with NSTE-ACS were more likely to have ≥2 long-term conditions *c.f.* those with STEMI (72.7 vs 53.0%; *P* < 0.001).^3^^,^^50^ In the NSTE-ACS subgroup, patients with multimorbidity were older (80.3 vs 78.7 years; *P* < 0.001) but were *not* more likely to be female (43.4 vs 43.2%; *P* > 0.05). In the STEMI subgroup, patients with multimorbidity were also older (80.5 vs 78.6 years; *P* < 0.001) but *were* more likely to be female (45.0% vs 42.1%; *P* < 0.001). Patients with multimorbidity had greater BMI (NSTE-ACS: 26.3 vs 25.7 kg/m^2^; *P* < 0.001 and STEMI: 26.1 vs 25.5 kg/m^2^; *P* < 0.001) but were less likely to be active smokers (NSTE-ACS: 8.9 vs 11.3; *P* < 0.001 and STEMI: 11.8 vs 14.7; *P* < 0.001). Among 302 patients hospitalized with ACS in Vietnam, those with multimorbidity tended to be older and presented with NSTE-ACS rather than STEMI.^63^ In 1,488 adults aged ≥65 years with NSTE-ACS in Australia, patients with ≥2 long-term conditions were older (80.3 vs 79.2 years; *P* = 0.034) but were no more likely to be female (36.6 vs 38.5; *P* > 0.05) vs those with <2 long-term conditions.^65^ Those with multimorbidity were more likely to have had previous PCI (27.8 vs 11.9%; *P* < 0.001) and CABG (14.2 vs 7.4%; *P* < 0.001) but were no more likely to live in residential care (2.5 vs 2.0%; *P* > 0.05).^65^

## Multimorbidity, clinical management, and outcomes of ACS

Thirty studies that reported at least one secondary outcome by multimorbidity status were identified and screened for risk of bias (6 very high risk,^41^^,^^42^^,^^45^, ^46^, ^47^^,^^52^^,^^69^ 14 high risk,^3^^,^^5^^,^^50^^,^^53^, ^54^, ^55^^,^^57^^,^^60^^,^^65^^,^^66^^,^^70^, ^71^, ^72^, ^73^^,^^77^^,^^78^ 9 some concerns,^44^^,^^48^^,^^49^^,^^61^^,^^63^^,^^64^^,^^74^, ^75^, ^76^ and only 2 low risk^4^^,^^79^) (Supplemental Table 5, Supplemental Figure 2).

### Invasive management of ACS

Inhomogeneity of the 10 studies that reported the association between pre-existing multimorbidity and ACS treatment precluded quantitative synthesis.^3^^,^^41^^,^^42^^,^^44^^,^^46^^,^^50^^,^^63^^,^^64^^,^^75^, ^76^, ^77^^,^^79^ Five studies suggested that patients with pre-existing multimorbidity were less likely to undergo routine invasive management.^3^^,^^41^^,^^42^^,^^50^^,^^75^^,^^76^^,^^79^ This was the case in the TIGRIS (long-Term rIsk, clinical manaGement, and healthcare Resource utilisation of stable coronary artery dISease) registry (n = 5,132; 84.6% vs 91.9%; *P* < 0.0001)^41^^,^^42^ and SWEDEHEART (for both NSTE-ACS: 49.7% vs 70.1%, *P* < 0.001 and STEMI 70.4 vs 86.5%; *P* < 0.001).^3^^,^^50^ An Australia-based study of patients with high-risk NSTE-ACS supported these findings (n = 16,771; 48.2% vs 67.4%) but suggested that those with pre-existing multimorbidity were more likely to undergo CABG (13.9% vs 5.4%).^75^^,^^76^ This was also observed in a retrospective analysis of nationally representative U.S. administrative data (n = 6,613,623), in which those with multimorbidity were less likely to undergo angiography (57.6% vs 70.5%; *P* < 0.001) and PCI (34.7% vs 52.3%; *P* < 0.001) but more likely to undergo CABG (9.5% vs 6.2%; *P* < 0.001).^79^ Two small studies (n = 302 and 120), both originating from Vietnam, reported a null association between pre-existing multimorbidity and invasive management.^63^^,^^64^

### Pharmacological management of ACS

Patients with multiple pre-existing long-term conditions were less likely to receive guideline-directed antiplatelet pharmacotherapy, a high-potency statin, an angiotensin-converting enzyme inhibitor or angiotensin II receptor blocker and a β-blocker—but were more likely to receive oral anticoagulation—in the post-MI period.^3^^,^^41^^,^^42^^,^^44^^,^^50^^,^^77^ This is supported from data reported from the TIGRIS registry,^41^^,^^42^ the SPUM-ACS (Special Program University Medicine-Acute Coronary Syndromes study) registry,^44^ SWEDEHEART,^3^^,^^50^ and the BleeMACS (Bleeding complications in a Multicenter registry of patients discharged after an Acute Coronary Syndrome) registry.^77^ A single, small study reported no difference in the receipt of secondary preventative medication by multimorbidity status.^77^

### All-cause mortality

**Figure 4:**
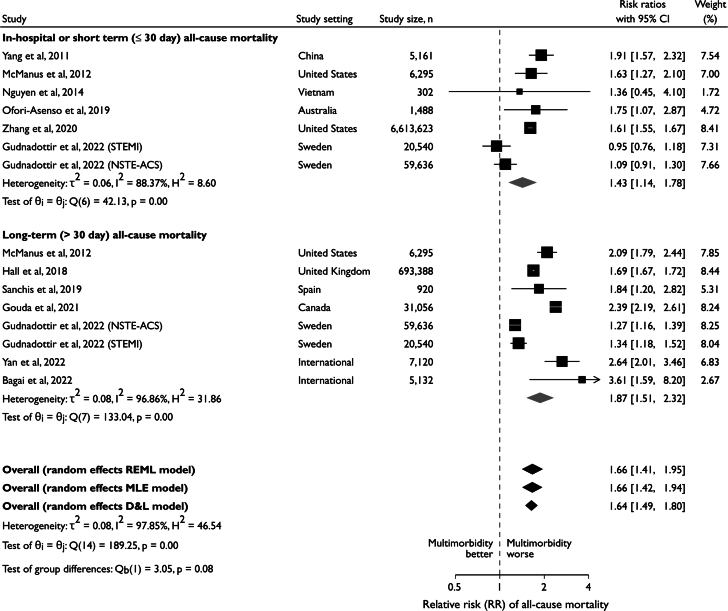
**Association Between Pre-Existing Multimorbidity, Short and Longer Term All-Cause Mortality** θ = effect size; D&L = DerSimonian and Laird; MLE = maximum likelihood estimation; NSTE-ACS = non–ST-segment elevation acute coronary syndrome; REML = restricted maximum likelihood; STEMI = ST-segment elevation myocardial infarction.

**Figure 5:**
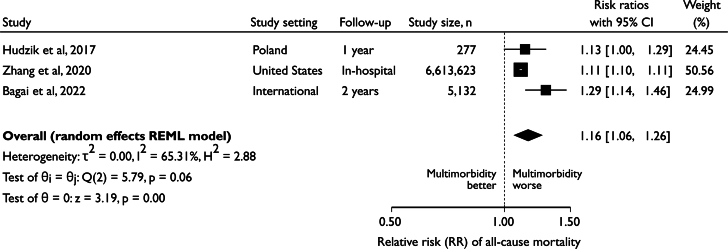
**The Association Between Multimorbidity Burden and All-Cause Mortality** The increased risk per additional long-term condition is shown. Abbreviations as in Figure 4.

Eleven studies reported the association of pre-existing multimorbidity with post-ACS all-cause mortality and were included in meta-analysis (Figure 4).^3^^,^^4^^,^^34^^,^^41^^,^^42^^,^^49^^,^^50^^,^^61^^,^^63^^,^^65^^,^^66^^,^^77^, ^78^, ^79^ Pre-existing multimorbidity was associated with increased all-cause mortality, with similar pooled effect sizes for short-term mortality (≤30 days follow-up; RR: 1.43; 95% CI: 1.14-1.78; *P* < 0.001; 6 studies^3^^,^^50^^,^^61^^,^^63^^,^^65^^,^^78^^,^^79^) and longer-term mortality (>30 days follow-up; RR: 1.87; 95% CI: 1.51-2.32; *P* for interaction = 0.08; 7 studies^3^^,^^4^^,^^34^^,^^41^^,^^42^^,^^49^^,^^50^^,^^61^^,^^66^^,^^77^). Substantial heterogeneity was noted (I^2^_short-term_ = 88.4, I^2^_longer-term_ = 96.7%; I^2^_overall_ = 97.9%). A sensitivity analysis was performed, which limited meta-analysis to per-study reported HRs. This demonstrated qualitatively similar results for both short-term and long-term mortality. Subgroup analysis and meta-regression did not identify study-level determinants of this heterogeneity. Meta-analysis of studies reporting the impact of multimorbidity *burden* demonstrated that every additional condition led to a 16% greater risk of all-cause mortality (RR: 1.16; 95% CI: 1.06-1.26; *P* < 0.01) (Figure 5).^41^^,^^42^^,^^53^^,^^54^^,^^79^ The symmetrical nature of the funnel plot (Supplemental Figure 4A) and nonsignificance of Begg’s and Egger’s test (*P* = 0.92 and 0.41, respectively) excluded major publication bias. The deterministic impact of any single large study was refuted (Supplemental Figures 4B and 4C).

Sixteen studies reported one or more additional clinical outcomes—including MACE, index hospitalization length of stay, unplanned hospital readmission, or health care costs—which were included in a narrative review.^3^^,^^41^^,^^42^^,^^44^^,^^48^, ^49^, ^50^^,^^64^^,^^65^^,^^69^, ^70^, ^71^, ^72^, ^73^, ^74^^,^^77^^,^^79^ The main findings are summarized below— a full narrative exploration of each outcome is included in the Supplemental Results section.

### Major adverse cardiovascular events

Overall, patients with pre-existing multimorbidity at ACS presentation experienced greater rates of MACE post-ACS both over the short and longer term, compared with those without pre-existing multimorbidity.^41^^,^^42^^,^^44^^,^^49^^,^^57^^,^^74^^,^^77^^,^^79^

### Length of stay, health care costs, and unplanned readmissions

Pre-existing multimorbidity was associated with: 1) greater length of stay during the index ACS hospitalization^3^^,^^50^^,^^65^^,^^70^, ^71^, ^72^, ^73^; 2) greater subsequent rates of unplanned readmission to hospital^3^^,^^50^^,^^70^^,^^73^; and 3) greater health care–associated costs, both during the index hospitalization for ACS,^79^ and in the year following ACS diagnosis.^48^

### Multimorbidity and patient-reported outcome measures

Five studies reported the association of multimorbidity with one or more patient-reported outcome measures in the post-ACS setting.^5^^,^^45^^,^^47^^,^^52^^,^^60^ Pre-existing multimorbidity at the time of MI diagnosis was associated with an adverse impact on quality of life,^5^ fatigue, physical activity and vital exhaustion,^45^^,^^52^^,^^60^ and life chaos.^47^

### Secular trends in multimorbidity prevalence

All studies that evaluated serial estimates of multimorbidity prevalence over time reported that the prevalence of pre-existing multimorbidity in those presenting with ACS increased year-on-year.^43^^,^^61^^,^^65^^,^^68^^,^^73^^,^^79^

## Discussion

### Summary of key findings

The prevalence of multimorbidity in individuals presenting with ACS was 46.6% in this meta-analysis. The prevalence of multimorbidity was heterogeneous across the included studies: study-level determinants included the number of long-term conditions studied (greater prevalence in studies that reported more LTCs) and study setting (greater prevalence in studies from high vs low- to middle-income countries). Pre-existing multimorbidity was associated with a 43% increased risk of 30-day all-cause mortality and 87% increased risk of longer-term mortality post-ACS, respectively. Each long-term condition present prior to ACS was associated with a 16% greater risk of all-cause mortality, post-ACS.

Given inhomogeneity of reporting across the studies, quantitative meta-analysis was not possible for all outcomes. In our narrative synthesis, individual studies reported pre-existing multimorbidity to be associated with older age, NSTEMI, previous cardiovascular procedures, and a greater BMI. In addition to an increased risk of post-ACS all-cause mortality, patients with multiple pre-existing long-term conditions were also more likely to experience other adverse outcomes, including MACE, longer length of stay in hospital, and unplanned readmission. Those with multiple long-term conditions were more likely to have greater health care–associated expenditure and to report worse quality of life following an ACS. Patients with multimorbidity at the time of ACS were less likely to receive standard guideline-directed care in the post-ACS period, including routine invasive management (coronary angiography ± PCI), dual antiplatelet therapy, a high-potency statin, an angiotensin-converting enzyme inhibitor or angiotensin II receptor blocker and a β-blocker but were more likely to undergo CABG and have an indication for oral anticoagulation and diuretic therapy. The effect of multimorbidity on referral to (and uptake of) cardiac rehabilitation in the post-ACS setting gave conflicting results (in 2 small studies, conducted in different health care settings).

### Integration of findings with wider literature

Patients with pre-existing multimorbidity may be less likely to receive guideline-recommended treatment in the context of ACS for several reasons. Firstly, there may be a perception among clinicians that there is a lack of evidence to support the universal application of relevant clinical guidelines, or a lack of proven safety and efficacy for specific interventions for these patients. There are very few comorbidity-based absolute contraindications to guideline-directed post-ACS therapies. Secondly, there may be concern that those with multiple long-term conditions are more likely to experience a greater risk of complications and adverse reactions to established ACS treatments (due to physiological vulnerability or interactions with pre-existing medications). Unfortunately, many of the landmark trials that established the safety and efficacy of treatments that make up contemporary ACS management pathways excluded patients with multiple long-term conditions^7^ (with a few notable exceptions, including the recently reported SENIOR-RITA [Older Patients with Non–ST-Segment Elevation Myocardial Infarction Randomized Interventional Treatment] trial^81^). As a consequence, clinical guidelines often remain agnostic on how these treatments should be applied to those with multimorbidity and/or significant frailty. At present, a person-centered paradigm of shared decision-making (which embraces this inherent uncertainty) is advised.^10^

The prevalence of multimorbidity in those with ACS was greater than that reported by a recent meta-analyses of multimorbidity in community settings (37.2%^82^). This was expected: patients with ACS are more likely to have long-term conditions that increase cardiovascular risk (such as hypertension and diabetes mellitus). To date, there have been few studies that have sought to ascertain the prevalence and impact of multimorbidity in hospitalized groups. In one study of 2.2 million Swiss inpatients, multimorbidity was present in 79.7% and was associated with greater in-hospital mortality, length of stay, and 1-year all-cause readmissions.^83^ A number of ongoing studies are seeking to improve the ascertainment and actionability of multimorbidity in hospitalized patients.^84^

Despite a growing body of literature in this field, significant evidence gaps remain. Despite the clear association of pre-existing multimorbidity with adverse outcomes in the post-ACS setting, it remains unclear how knowledge of a patient’s multimorbidity status should inform clinical decision-making in this context. Recent guidelines are unhelpful in this regard: they emphasize the importance of shared decision-making and patient-centered care, without making specific treatment recommendations.^10^ A recent survey highlights that there is a significant clinical need for focused and actionable guidelines among cardiologists, especially for patients with multimorbidity.^85^

To date, most studies have evaluated the prevalence and impact of a definition-based, binary multimorbidity status in the context of ACS. However, multimorbidity is a heterogeneous clinical phenotype: 2 patients presenting with ACS with multiple pre-existing long-term conditions are likely to be very different. Future research must use more advanced methods to produce actionable insights for defined subpopulations of patients with multimorbidity, in order to target individuals for enhanced follow-up and specific interventions (or more conservative management) in the post-ACS period. Such methods should focus on the identification of combinations of long-term conditions that are causally implicated in the development of adverse outcomes. A more granular understanding of which patients with multiple long-term conditions are at greatest risk may enable greater personalization of clinical follow-up and may enable the identification of shared risk factors or pathways that may be targeted by novel therapeutic strategies to improve patient outcomes.

### Strengths and limitations

This study used a systematic and preregistered methodology to identify and summarize the current literature with regard to multimorbidity and ACS. However, there are some notable limitations. Firstly, many of the studies identified during the literature search demonstrated a high risk of bias. The application of stringent exclusion criteria (decided a priori) ensured that only methodologically robust studies, reporting an unbiased estimate of multimorbidity prevalence at MI presentation, were included in the quantitative synthesis. The most common reason for exclusion was the presence of selection bias in selecting the sample frame (restrictive inclusion criteria, the use of convenience samples, or the need for individual-level informed consent), uncontrolled confounding and biased reporting of results.

Secondly, despite exclusion of those studies at high risk of bias, the studies included in this study remained heterogeneous. Meta-regression analyses suggested that the number of LTCs chosen for inclusion and the setting of the studies (high vs low- to middle-income countries) were responsible for some of this heterogeneity. The long-term conditions included in each study may be another contributing factor. To improve comparability, future studies should report a consistent definition of multimorbidity, operationalized using a uniform, standardized list of long-term conditions. This recommendation is in line with a recent systematic review which identified a similar effect in meta-regression analysis on 193 studies that reported the prevalence of multimorbidity.^86^ Subsequently, a Delphi study has established a consensus regarding which long-term conditions should be included in future studies, from a wide range of stakeholders.^87^

Thirdly, although most cardiovascular studies report the prevalence of individual comorbidities at baseline, few report the proportion of subjects with multiple long-term conditions. As such, an exhaustive search strategy (with extensive review of full-text studies) was required to identify those studies that reported counts of pre-existing long-term conditions in the context of ACS. Changes to reporting guidelines to encourage such data to be routinely reported may enable future reviews to be more efficient in this regard. This would be of particular benefit in the context of clinical trials: enabling assessment of whether the multimorbidity burden of those included are comparable to real-world patient cohorts.

Finally, many of the included studies reported on data collected from a single hospitalization episode, which is unlikely to be a comprehensive record of an individual’s long-term conditions and may lead to possible underestimation of multimorbidity.^88^^,^^89^ The integration of secondary care–derived data with external data sources (which ideally spans the entire life-course of an individual, such as primary care or insurance claims data) is required to adequately capture the past medical history of an individual. Finally, a small proportion of patients with ACS may not present to hospital (or may die en route to hospital). These patients may be expected to have a greater multimorbidity burden than those included in an “in-hospital” analysis. Therefore, ideally, an estimation of the prevalence of multimorbidity in ACS should also include these patient groups (eg, by incorporating community death certificate data).

## Conclusions

Multiple long-term conditions are common in the context of ACS and associated with reduced use of guideline-directed treatment and a range of adverse clinical outcomes. The studies included in this review had a high risk of bias and significant heterogeneity in how they defined and operationalized multimorbidity. Going forward, consistent definitions of multimorbidity must be applied to enable greater comparability across studies. At present, it remains unclear how knowledge of a patient’s multimorbidity status should be taken into account when making clinical decisions in the context of ACS. Future research should focus on the identification of specific patterns and accumulation of long-term conditions that are most associated with adverse outcomes, in order to translate into novel therapeutic strategies, treatment pathways, and other insights that can improve shared clinical decision-making in the post-ACS context.

Perspectives **COMPETENCY IN SYSTEMS-BASED PRACTICE:** Individuals with ACS commonly have multiple pre-existing long-term conditions, the presence of which are associated with a range of adverse post-ACS clinical outcomes. Care models that integrate traditional systems-based approaches to health care delivery and equally value generalist and specialist clinical input are required to optimize the care of this population.

**COMPETENCY IN PATIENT CARE AND PROCEDURAL SKILLS:** A holistic, person-centered approach to pre-existing long-term conditions is required for those presenting with ACS. Shared decision-making, based on current evidence-based clinical guidelines, must inform invasive and pharmacological management, recognizing uncertainty where it prevails. Clinical management should be informed by multidisciplinary input, addressing social determinants of health and ensuring equitable use of resources.

**TRANSLATIONAL OUTLOOK:** This study, which reported a systematic review and meta-analysis of the prevalence and impact of pre-existing multimorbidity in individuals presenting with ACS, found that: 1) multiple pre-existing long-term conditions were common at ACS presentation; 2) pre-existing multimorbidity was associated with reduced guideline-directed treatment; and 3) multimorbidity was associated with adverse post-ACS clinical outcomes. Given demographic trends, the prevalence of long-term conditions among those presenting with ACS is likely to increase further, placing added pressure on health systems to manage complex combinations of conditions effectively and increasing the need for integrated, long-term care strategies. Strategies to identify patterns of multimorbidity associated with the greatest risk (or benefit from specific interventions) are required.

## Funding support and author disclosures

This work was funded by a 10.13039/100010269Wellcome Trust 4ward North Clinical Research Training Fellowship (grant numbers 227498/Z/23/Z, R127002) and a 10.13039/100010269Wellcome Trust Sir Henry Wellcome Postdoctoral Fellowship scheme (206470/Z/17/Z). The authors have reported that they have no relationships relevant to the contents of this paper to disclose.
