# Supplementary material for: Multimorbidity in Acute Coronary Syndrome: A Systematic Review and Meta-Analysis
Source: JACC Adv. 2025 Jul 22;4(8):102006. doi: 10.1016/j.jacadv.2025.102006 (PMC12308028; doi:10.1016/j.jacadv.2025.102006)
Supplement: Supplemental Material [file mmc1.docx]

**Supplemental Table 1** | Meta-analysis Of Observational Studies in Epidemiology (MOOSE) checklist.

| **Item No** | **Recommendation** | **Reported on Page No** |
| --- | --- | --- |
| Reporting of background should include | | |
| 1 | Problem definition | 4, 5 |
| 2 | Hypothesis statement | 4, 5 |
| 3 | Description of study outcome(s) | 7 |
| 4 | Type of exposure or intervention used | 6, 7 |
| 5 | Type of study designs used | 6 |
| 6 | Study population | 6 |
| Reporting of search strategy should include | | |
| 7 | Qualifications of searchers (eg, librarians and investigators) | 8 |
| 8 | Search strategy, including time period included in the synthesis and key words | 7, Supp 6 |
| 9 | Effort to include all available studies, including contact with authors | N/A |
| 10 | Databases and registries searched | 8 |
| 11 | Search software used, name and version, including special features used (eg, explosion) | 8 |
| 12 | Use of hand searching (eg, reference lists of obtained articles) | 8 |
| 13 | List of citations located and those excluded, including justification | N/A |
| 14 | Method of addressing articles published in languages other than English | 8 |
| 15 | Method of handling abstracts and unpublished studies | 8 |
| 16 | Description of any contact with authors | N/A |
| Reporting of methods should include | | |
| 17 | Description of relevance or appropriateness of studies assembled for assessing the hypothesis to be tested | 6, 7 |
| 18 | Rationale for the selection and coding of data (eg, sound clinical principles or convenience) | 6, 7 |
| 19 | Documentation of how data were classified and coded (eg, multiple raters, blinding and interrater reliability) | 8 |
| 20 | Assessment of confounding (eg, comparability of cases and controls in studies where appropriate) | 7, 8 |
| 21 | Assessment of study quality, including blinding of quality assessors, stratification or regression on possible predictors of study results | 7, 8 |
| 22 | Assessment of heterogeneity | 9 |
| 23 | Description of statistical methods (eg, complete description of fixed or random effects models, justification of whether the chosen models account for predictors of study results, dose-response models, or cumulative meta-analysis) in sufficient detail to be replicated | 9, 10 |
| 24 | Provision of appropriate tables and graphics | Tab 1, 2, Supp 7–15 |
| Reporting of results should include | | |
| 25 | Graphic summarizing individual study estimates and overall estimate | Fig 1 – 3 |
| 26 | Table giving descriptive information for each study included | Tab 1 |
| 27 | Results of sensitivity testing (eg, subgroup analysis) | 13, Fig 2, Supp 14 |
| 28 | Indication of statistical uncertainty of findings | 13, 16, 17 |
| Reporting of discussion should include | | |
| 29 | Quantitative assessment of bias (eg, publication bias) | 15, Supp 7 – 11 |
| 30 | Justification for exclusion (eg, exclusion of non-English language citations) | Supp 11 |
| 31 | Assessment of quality of included studies | Supp 7 – 11 |
| Reporting of conclusions should include | | |
| 32 | Consideration of alternative explanations for observed results | 20 – 23 |
| 33 | Generalization of the conclusions (ie, appropriate for the data presented and within the domain of the literature review) | 23 |
| 34 | Guidelines for future research | 23 |
| 35 | Disclosure of funding source | 1 |

*From*: Stroup DF, Berlin JA, Morton SC, et al, for the Meta-analysis Of Observational Studies in Epidemiology (MOOSE) Group. Meta-analysis of Observational Studies in Epidemiology. A Proposal for Reporting. *JAMA*. 2000;283(15):2008-2012. doi: 10.1001/jama.283.15.2008.

**Supplemental Table 2** | Preferred Reporting Items for Systematic Reviews and Meta-Analyses (PRISMA) checklist.

| **Section and Topic** | **Item #** | **Checklist item** | **Location where item is reported** |
| --- | --- | --- | --- |
| **TITLE** | | |  |
| Title | 1 | Identify the report as a systematic review. | 1 |
| **ABSTRACT** | | |  |
| Abstract | 2 | See the PRISMA 2020 for Abstracts checklist. | 2 |
| **INTRODUCTION** | | |  |
| Rationale | 3 | Describe the rationale for the review in the context of existing knowledge. | 4, 5 |
| Objectives | 4 | Provide an explicit statement of the objective(s) or question(s) the review addresses. | 4, 5 |
| **METHODS** | | |  |
| Eligibility criteria | 5 | Specify the inclusion and exclusion criteria for the review and how studies were grouped for the syntheses. | 6, 7 |
| Information sources | 6 | Specify all databases, registers, websites, organisations, reference lists and other sources searched or consulted to identify studies. Specify the date when each source was last searched or consulted. | 8 |
| Search strategy | 7 | Present the full search strategies for all databases, registers and websites, including any filters and limits used. | Supp 6 |
| Selection process | 8 | Specify the methods used to decide whether a study met the inclusion criteria of the review, including how many reviewers screened each record and each report retrieved, whether they worked independently, and if applicable, details of automation tools used in the process. | 8 |
| Data collection process | 9 | Specify the methods used to collect data from reports, including how many reviewers collected data from each report, whether they worked independently, any processes for obtaining or confirming data from study investigators, and if applicable, details of automation tools used in the process. | 8 |
| Data items | 10a | List and define all outcomes for which data were sought. Specify whether all results that were compatible with each outcome domain in each study were sought (e.g. for all measures, time points, analyses), and if not, the methods used to decide which results to collect. | 7 |
|  | 10b | List and define all other variables for which data were sought (e.g. participant and intervention characteristics, funding sources). Describe any assumptions made about any missing or unclear information. | 7 |
| Study risk of bias assessment | 11 | Specify the methods used to assess risk of bias in the included studies, including details of the tool(s) used, how many reviewers assessed each study and whether they worked independently, and if applicable, details of automation tools used in the process. | 8 |
| Effect measures | 12 | Specify for each outcome the effect measure(s) (e.g. risk ratio, mean difference) used in the synthesis or presentation of results. | 9 |
| Synthesis methods | 13a | Describe the processes used to decide which studies were eligible for each synthesis (e.g. tabulating the study intervention characteristics and comparing against the planned groups for each synthesis (item #5)). | 6, 7 |
|  | 13b | Describe any methods required to prepare the data for presentation or synthesis, such as handling of missing summary statistics, or data conversions. | 9 |
|  | 13c | Describe any methods used to tabulate or visually display results of individual studies and syntheses. | 9 |
|  | 13d | Describe any methods used to synthesize results and provide a rationale for the choice(s). If meta-analysis was performed, describe the model(s), method(s) to identify the presence and extent of statistical heterogeneity, and software package(s) used. | 9 |
|  | 13e | Describe any methods used to explore possible causes of heterogeneity among study results (e.g. subgroup analysis, meta-regression). | 9 |
|  | 13f | Describe any sensitivity analyses conducted to assess robustness of the synthesized results. | 13, 15 |
| Reporting bias assessment | 14 | Describe any methods used to assess risk of bias due to missing results in a synthesis (arising from reporting biases). | 9 |
| Certainty assessment | 15 | Describe any methods used to assess certainty (or confidence) in the body of evidence for an outcome. | 9 |
| **RESULTS** | | |  |
| Study selection | 16a | Describe the results of the search and selection process, from the number of records identified in the search to the number of studies included in the review, ideally using a flow diagram. | 11, Fig 1, Supp 12 |
|  | 16b | Cite studies that might appear to meet the inclusion criteria, but which were excluded, and explain why they were excluded. | N/A |
| Study characteristics | 17 | Cite each included study and present its characteristics. | Table 1 |
| Risk of bias in studies | 18 | Present assessments of risk of bias for each included study. | Supp 8 – 11 |
| Results of individual studies | 19 | For all outcomes, present, for each study: (a) summary statistics for each group (where appropriate) and (b) an effect estimate and its precision (e.g. confidence/credible interval), ideally using structured tables or plots. | 13, 16, 17, Figures 2, 3, 4, 5 |
| Results of syntheses | 20a | For each synthesis, briefly summarise the characteristics and risk of bias among contributing studies. | 15 |
|  | 20b | Present results of all statistical syntheses conducted. If meta-analysis was done, present for each the summary estimate and its precision (e.g. confidence/credible interval) and measures of statistical heterogeneity. If comparing groups, describe the direction of the effect. | 13, 16, 17, Figures 2, 3, 4, 5 |
|  | 20c | Present results of all investigations of possible causes of heterogeneity among study results. | 13, 14 |
|  | 20d | Present results of all sensitivity analyses conducted to assess the robustness of the synthesized results. | Suppl 15 |
| Reporting biases | 21 | Present assessments of risk of bias due to missing results (arising from reporting biases) for each synthesis assessed. | Suppl 15 |
| Certainty of evidence | 22 | Present assessments of certainty (or confidence) in the body of evidence for each outcome assessed. | Suppl 15 |
| **DISCUSSION** | | |  |
| Discussion | 23a | Provide a general interpretation of the results in the context of other evidence. | 19 |
|  | 23b | Discuss any limitations of the evidence included in the review. | 22, 23 |
|  | 23c | Discuss any limitations of the review processes used. | 22, 23 |
|  | 23d | Discuss implications of the results for practice, policy, and future research. | 23 |
| **OTHER INFORMATION** | | |  |
| Registration and protocol | 24a | Provide registration information for the review, including register name and registration number, or state that the review was not registered. | 3 |
|  | 24b | Indicate where the review protocol can be accessed, or state that a protocol was not prepared. | 6 |
|  | 24c | Describe and explain any amendments to information provided at registration or in the protocol. | N/A |
| Support | 25 | Describe sources of financial or non-financial support for the review, and the role of the funders or sponsors in the review. | 1 |
| Competing interests | 26 | Declare any competing interests of review authors. | 1 (none to declare). |
| Availability of data, code and other materials | 27 | Report which of the following are publicly available and where they can be found: template data collection forms; data extracted from included studies; data used for all analyses; analytic code; any other materials used in the review. | N/A |

*From:*  Page MJ, McKenzie JE, Bossuyt PM, Boutron I, Hoffmann TC, Mulrow CD, et al. The PRISMA 2020 statement: an updated guideline for reporting systematic reviews. BMJ 2021;372:n71. doi: 10.1136/bmj.n71

**Supplemental Table 3** | Medline and Embase query, conducted via the Ovid interface.

| **Concept 1: ACS-related terms** |
| --- |
| Myocardial Infarction/ |
| ((myocardial adj2 infarct*) or (myocardium adj2 infarct*)).tw |
| Acute Coronary Syndrome/ |
| acute coronary syndrome.tw |
| STEMI/ |
| (ST elevation or ST segment elevation or STEMI).tw |
| NSTEMI/ |
| (non ST elevation or non ST segment elevation or NSTEMI).tw |
| **Concept 2: Multimorbidity-related terms** |
| Multimorbidity/ |
| (multimorbid* or multi-morbid*).tw |
| (multidisease? or multicondition? or ((multi* or multiple) adj2 ((morbid* or ill* or disease? or condition? or syndrom* or disorder? or chronic) not (regression or imputation)))).tw |
| ((multiple or multi*) adj ((comorbid* or co-morbid*) or ((chronic* or long-term) adj (disease? or ill* or care or condition? or disorder* or health* or medication* or syndrom* or symptom*)))).tw |
| ((Charlson or Elixhauser or co-morbid* or comorbid*) adj (index* or indice* or indicator* or score* or scale* or tool* or test* or model* or phenotyp* or criteri* or marker* or method* or instrument* or assess* or exam* or evaluat* or measure* or screen* or diagnos* or detect* or identif*)).tw |

Symbols and abbreviations: “/”: subject heading search, “*”: truncation (identifies variant endings for the stem word), “?”: wildcard (allows a different character (or no character) to identify variant spellings of words, “.tw”: limit to title and abstract fields. Notes: each term within a concept is combined with the ‘OR’ operator, then concepts are combined using the ‘AND’ operator.

**Supplemental Table 4 |** Risk of bias of studies included in the synthesis of multimorbidity prevalence in patients with ACS (JBI prevalence checklist).

| **Study, year [refs]** | **Responses to JBI Questions** | | | | | | | | | **Overall Decision** | **Justification** |
| --- | --- | --- | --- | --- | --- | --- | --- | --- | --- | --- | --- |
|  | **1** | **2** | **3** | **4** | **5** | **6** | **7** | **8** | **9** |  |  |
|  | Appropriate sample frame used | Appropriate recruitment/  inclusion criteria | Sample size is adequate | Detailed description (subjects/setting) | Sufficient coverage of sample | Valid method of identifying condition | Condition measured in reliable way | Appropriate statistical analysis | Adequate response rate (recruitment) |  |  |
| Alsawas,  2019[1] |  |  |  |  |  |  |  |  |  | Include | Unselected hospitalised patients with incident MI; reports proportion with multimorbidity. |
| Attar,  2022[2] |  |  |  |  |  |  |  |  |  | Include | Unselected hospitalised patients with incident MI; reports proportion with multimorbidity. |
| Bagai,  2022[3, 4] |  |  |  |  |  |  |  |  |  | Exclude | Recruitment at 1-3 y post-MI: recruited survivors of MI, likely to be less multimorbid. |
| Canivell,  2018[5] |  |  |  |  |  |  |  |  |  | Include | Hospitalised patients with incident MI; some exclusions, reports multimorbid proportion. |
| Chen,  2015[6] |  |  |  |  |  |  |  |  |  | Exclude | Selected patients with incident MI surviving hospitalisation, likely to be less multimorbid. |
| Crane,  2005[7] |  |  |  |  |  |  |  |  |  | Exclude | Convenience sample (54% response); selected group (women aged ≥ 65), 6-12 m post-MI. |
| Dunn,  2009[8] |  |  |  |  |  |  |  |  |  | Exclude | Selected patients with incident MI surviving hospitalisation (< 73% response rate to participate). |
| Ganasegeran,  2018[9] |  |  |  |  |  |  |  |  |  | Exclude | Selected patients with incident MI surviving to OP follow-up; only 3 conditions evaluated. |
| Ghushchyan,  2015[10] |  |  |  |  |  |  |  |  |  | Exclude | Patient that survived ACS and participated in MEPS survey; unknown comorbidities included. |
| Gouda,  2021[11] |  |  |  |  |  |  |  |  |  | Include | Unselected hospitalised patients with incident MI; reports proportion with multimorbidity. |
| Gudnadottir,  2022[12, 13] |  |  |  |  |  |  |  |  |  | Include* | Unselected hospitalised patients aged ≥ 70 y with incident MI; reports multimorbid proportion. |
| Gutacker,  2015[14] |  |  |  |  |  |  |  |  |  | Include | Unselected hospitalised patients with incident MI; reports proportion with multimorbidity. |
| Hall,  2018[15, 16] |  |  |  |  |  |  |  |  |  | Include | Unselected hospitalised patients with incident MI; reports proportion with multimorbidity. |
| Horne,  2019[17] |  |  |  |  |  |  |  |  |  | Exclude | Selected patients, aged ≥ 65 with incident MI surviving 6-8 m; likely to be less multimorbid. |
| Hudzik,  2017[18, 19] |  |  |  |  |  |  |  |  |  | Exclude | Selected (diabetic) patients, with incident MI; reports proportion with multimorbidity. |
| Jain,  2022[20] |  |  |  |  |  |  |  |  |  | Include* | Semi-selected patients (≥ 66 and < 90 y; Medicare cover; excluded metastasis and dementia). |
| Johnman,  2012[21] |  |  |  |  |  |  |  |  |  | Exclude | Selected hospitalised patients (undergoing invasive management for STEMI), |
| Kim,  2023[83] |  |  |  |  |  |  |  |  |  | Exclude | Selected (female) hospitalised patients with incident ACS; only 5 conditions evaluated. |
| King,  2021[22, 23] |  |  |  |  |  |  |  |  |  | Exclude | Unselected hospitalised patients with incident MI; sample size only n = 223 (< 288 threshold) |
| McGowan,  2004[24] |  |  |  |  |  |  |  |  |  | Exclude | Selected hospitalised patients surviving MI (< 80, no previous MI, no cognitive impairment). |
| McManus,  2012[25] |  |  |  |  |  |  |  |  |  | Exclude | Selected patients with incident MI surviving hospitalisation, likely to be less multimorbid. |

| **Study, year [refs]** | **Responses to JBI Questions** | | | | | | | | | **Overall Decision** | **Justification** |
| --- | --- | --- | --- | --- | --- | --- | --- | --- | --- | --- | --- |
|  | **Question 1** | **Question 2** | **Question 3** | **Question 4** | **Question 5** | **Question 6** | **Question 7** | **Question 8** | **Question 9** |  |  |
|  | Appropriate sample frame used | Appropriate recruitment/inclusion criteria | Sample size is adequate | Detailed description (subjects/setting) | Sufficient coverage of sample | Valid method of identifying condition | Condition measured in reliable way | Appropriate statistical analysis | Adequate response rate (recruitment) |  |  |
| Munyombwe,  2021[26] |  |  |  |  |  |  |  |  |  | Include | Hospitalised patients with first incident MI that consented to participate; initial response 97.5%. |
| Navathe,  2013[27] |  |  |  |  |  |  |  |  |  | Include* | Semi-selected patients (≥ 66 and < 90 y; Medicare cover, excluded those with LOS < 2 d). |
| Nguyen,  2014[28] |  |  |  |  |  |  |  |  |  | Include | Unselected hospitalised patients with incident first MI; reports proportion with multimorbidity. |
| Nguyen,  2020[29] |  |  |  |  |  |  |  |  |  | Exclude | Selected hospitalised patients aged ≥ 80 y; multiple exclusion criteria; sample size only n = 120 |
| Ofori-Asenso,  2019[30] |  |  |  |  |  |  |  |  |  | Include* | Unselected hospitalised patients aged ≥ 65 y with NSTEMI, reports multimorbid proportion. |
| Sanchis,  2019[31] |  |  |  |  |  |  |  |  |  | Include* | Hospitalised patients aged ≥ 65 y with NSTE-ACS that consented to participate. |
| Sanchis,  2021[32] |  |  |  |  |  |  |  |  |  | Include* | Hospitalised patients aged ≥ 65 y with NSTE-ACS that consented to participate. |
| Sun,  2020[33] |  |  |  |  |  |  |  |  |  | Include | Unselected hospitalised patients with incident MI; reports proportion with multimorbidity. |
| Tisminetzky,  2016a[34] |  |  |  |  |  |  |  |  |  | Exclude | Selected patients with incident MI surviving hospitalisation, likely to be less multimorbid. |
| Tisminetzky,  2016b[35] |  |  |  |  |  |  |  |  |  | Exclude | Selected patients with incident MI surviving 6 m post-discharge; likely to be less multimorbid. |
| Tisminetzky,  2018[36] |  |  |  |  |  |  |  |  |  | Include* | Hospitalised patients aged ≥ 65 y with incident acute MI; reports prevalence of multimorbidity. |
| Tisminetzky,  2019[37] |  |  |  |  |  |  |  |  |  | Include* | Hospitalised patients aged ≥ 65 y with incident acute MI; reports prevalence of multimorbidity. |
| Tisminetzky,  2021[38] |  |  |  |  |  |  |  |  |  | Exclude | Selected patients with incident MI surviving hospitalisation, likely to be less multimorbid. |
| Turner,  2020[39] |  |  |  |  |  |  |  |  |  | Include | Hospitalised patients with incident NSTE-ACS that consented to participate |
| Worrall-Carter,  2016a[40] |  |  |  |  |  |  |  |  |  | Include | Unselected hospitalised patients with incident NSTE-ACS; reports multimorbid proportion. |
| Worrall-Carter,  2016b[41] |  |  |  |  |  |  |  |  |  | Include | Unselected hospitalised patients with incident ACS; reports proportion with multimorbidity. |
| Yan,  2022[42] |  |  |  |  |  |  |  |  |  | Exclude | Consecutive hospitalised patients that consented to participate and survived to discharge. |
| Yang,  2011[43] |  |  |  |  |  |  |  |  |  | Include | Unselected hospitalised patients with incident MI; reports proportion with multimorbidity. |
| Zhang,  2020[44] |  |  |  |  |  |  |  |  |  | Include | Unselected hospitalised patients with incident ACS; reports proportion with multimorbidity. |
| Zykov,  2022[45] |  |  |  |  |  |  |  |  |  | Include | Unselected hospitalised patients with incident MI; reports proportion with multimorbidity. |

Classification of risk of bias based on the Joanna Briggs Institute Prevalence Checklist tool.[46, 47] Footnotes: *Included in a subgroup analysis of studies that restrict their analysis to the older population. Key – judgement:

Yes

No

Not applicable

Unclear

**Supplemental Table 5 |** Risk of bias of studies included in the synthesis of the effect of multimorbidity on outcomes in patients with ACS (ROBINS-E).

| **Study, year**  **[refs]** | **ROBINS-E Domains** | | | | | | | **Overall** |
| --- | --- | --- | --- | --- | --- | --- | --- | --- |
|  | **Domain 1** | **Domain 2** | **Domain 3** | **Domain 4** | **Domain 5** | **Domain 6** | **Domain 7** |  |
|  | Bias due to confounding | Bias arising from measurement of the exposure | Bias in selection of participants into the study | Bias due to post-exposure interventions | Bias due to missing data | Bias arising from measurement of the outcome | Bias in selection of the reported result |  |
| Bagai,  2022[3, 4] |  |  |  |  |  |  |  |  |
| Canivell,  2018[5] |  |  |  |  |  |  |  |  |
| Crane,  2005[7] |  |  |  |  |  |  |  |  |
| Dunn,  2009[8] |  |  |  |  |  |  |  |  |
| Ghushchyan,  2015[10] |  |  |  |  |  |  |  |  |
| Ganasegeran,  2018[9] |  |  |  |  |  |  |  |  |
| Gouda,  2021[11] |  |  |  |  |  |  |  |  |
| Gudnadottir,  2022[12, 13] |  |  |  |  |  |  |  |  |
| Hall,  2018[15, 16] |  |  |  |  |  |  |  |  |
| Horne,  2019[17] |  |  |  |  |  |  |  |  |
| Hudzik,  2017[18, 19] |  |  |  |  |  |  |  |  |
| Jain,  2022[20] |  |  |  |  |  |  |  |  |
| Kim,  2023[78] |  |  |  |  |  |  |  |  |
| McManus,  2012[25] |  |  |  |  |  |  |  |  |
| McGowan,  2004[24] |  |  |  |  |  |  |  |  |
| Munyombwe,  2021[26] |  |  |  |  |  |  |  |  |
| Nguyen,  2014[28] |  |  |  |  |  |  |  |  |
| Nguyen,  2020[29] |  |  |  |  |  |  |  |  |
| Ofori-Asenso,  2019[30] |  |  |  |  |  |  |  |  |
| Sanchis,  2019[31] |  |  |  |  |  |  |  |  |
| Tisminetzky,  2016a[34] |  |  |  |  |  |  |  |  |

| **Study, year**  **[refs]** | **ROBINS-E Domains** | | | | | | | **Overall** |
| --- | --- | --- | --- | --- | --- | --- | --- | --- |
|  | **Domain 1** | **Domain 2** | **Domain 3** | **Domain 4** | **Domain 5** | **Domain 6** | **Domain 7** |  |
|  | Bias due to confounding | Bias arising from measurement of the exposure | Bias in selection of participants into the study | Bias due to post-exposure interventions | Bias due to missing data | Bias arising from measurement of the outcome | Bias in selection of the reported result |  |
| Tisminetzky,  2016b[35] |  |  |  |  |  |  |  |  |
| Tisminetzky,  2018[36] |  |  |  |  |  |  |  |  |
| Tisminetzky,  2019[37] |  |  |  |  |  |  |  |  |
| Tisminetzky,  2021[38] |  |  |  |  |  |  |  |  |
| Turner,  2020[39] |  |  |  |  |  |  |  |  |
| Worrall-Carter,  2016a[40] |  |  |  |  |  |  |  |  |
| Worrall-Carter,  2016b[41] |  |  |  |  |  |  |  |  |
| Yan,  2022[42] |  |  |  |  |  |  |  |  |
| Yang,  2011[43] |  |  |  |  |  |  |  |  |
| Zhang,  2020[44] |  |  |  |  |  |  |  |  |

Classification of the risk of bias based on the ROBINS-E tool[48]. Abbreviations: ACS – acute coronary syndrome, MACE – major adverse cardiovascular events, MM - multimorbidity. Key – judgement:

Very high

High

Some concerns

Low

No information

Not applicable

**Supplemental Figure 1** | Full PRISMA (2020) flow diagram

Full-text reports excluded:

- Does not report multimorbidity (n = 2)
- Duplicate studies (n = 2)
- Selected sub-population of MI (n = 0)
- Insufficient information reported (n = 0)
- Does not focus on MI (n = 0)
- Entirely pre-2000 MI cohort (n = 0)
- Protocol for study only (n = 0)
- Other reason for exclusion (n = 0)

Note: these are not mutually exclusive; exclusion for multiple reasons was possible.

Full-text reports assessed for eligibility.

(n = 617)

Full-text reports excluded:

- Does not report multimorbidity (n = 362)
- Selected sub-population of MI (n = 46)
- Insufficient information reported (n = 42)
- Does not focus on MI (n = 40)
- Other reason for exclusion (n = 72)
- Entirely pre-2000 MI cohort (n = 14)
- Protocol for study only (n = 2)

Note: these are not mutually exclusive; exclusion for multiple reasons was possible.

Reports assessed for eligibility.

(n = 6)

Studies included in review.

(n = 41)

Reports of included studies

(n = 46)

**Included**

Records screened.

(n = 9,812)

Records excluded by title/abstract review.

(n = 9,192)

Full-text reports sought for retrieval.

(n = 620)

Reports not retrieved.

(n = 3)

**Screening**

Reports sought for retrieval.

(n = 8)

Reports not retrieved.

(n = 0)

Records identified from:

- Ovid Medline (n = 3,410)
- EMBASE (n = 8,037)
- Web of Science (n = 2,613)
- Cochrane CENTRAL (n = 0,686)
- Cochrane Syst. Rev. (n = 0,056)

Total records identified: (n = 14,802)

Removed *before screening*:

- Duplicates removed (n = 4,990)
- Records marked as ineligible by automation tools (n = 0)
- Records removed for other reasons (n = 0)

Records identified from:

- Reference screening (n = 5)
- Other systematic reviews (n = 3)
- Websites (n = 0)
- Organisations (n = 0)

**Identification of studies via databases and registers**

**Identification of studies via other methods**

**Identification**

**Supplemental Figure 2** | Summary of the overall risk of bias in the included studies, as assessed using the ROBINS-E tool.

ç
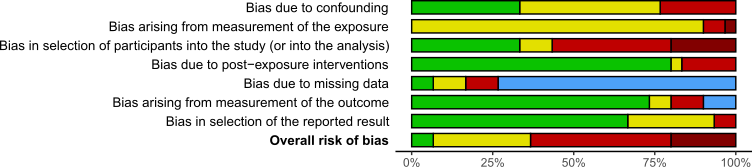


Very high

High

Some concerns

Low

No information

Not applicable

**Supplemental Figure 3** | Meta-regression bubble plots for the association of (a) mean age of sample, and (b) median year of study period, with the prevalence of multimorbidity.

(a) β = 0.004 (95% CI: -0.014 – 0.021); *p* = 0.700; R^2^ = 0%.

(b) β = 0.004 (95% CI: -0.014 – 0.021); *p* = 0.678; R^2^ = 0%.

Trend lines calculated using DerSimonian and Laird (inverse variance) weighting method.

**Supplemental Figure 4** | Diagnostic plots for the meta-analysis of effect of multimorbidity on all-cause mortality. (a) funnel plot; (b) Galbraith plot, and (c) a leave-one-out plot.

(a)

(b)

(c)

Abbreviations: θ – effect size, CI – confidence interval, REML – restricted maximum likelihood, se – standard error. se_j_ is estimated using the formula: $\sqrt{\sigma_{j}^{2}+\tau_{REML}^{2}}$.

**Supplemental Results**

**Major adverse cardiovascular events (MACE)**

In a UK-based study of 1,456 patients with NSTE-ACS, pre-existing multimorbidity was associated with a 1.76-fold increase in MACE (recurrent MI, ischaemic stroke or cardiovascular death) over 19 months of follow-up (HR 1.76, 95% CI 1.10 – 2.82)[39]. A study from Canada reported that those with 2 or ≥ 3 LTCs had a 1.57 and 2.61-fold risk of MACE (defined as death, recurrent MI or stroke) at three years post-MI (HR for 2 *vs.* 0 conditions: 1.57, 95% CI 1.47 – 1.68; HR for ≥ 3 *vs*. 0 conditions: 2.61, 95% CI 2.40 – 2.85). A well-powered, US-based study (*n* = 6,613,623) reported that for each additional LTC, the odds of an in-hospital MACE event (defined as death, stroke or a cardiac or vascular complication) were 11% greater (OR per additional comorbidity: 1.11, 95% CI 1.10 – 1.12)[44]. Similarly, the TIGRIS registry reported that for each additional long-term condition present at the time of MI, the risk of MACE (nonfatal MI, unstable angina, stroke or cardiovascular death) at 2 years increased by a third (RR per additional comorbidity: 1.33, 95% CI 1.25 - 1.42)[3, 4]. Similar associations were noted in two additional studies[5, 37].

**Length of stay**

Pre-existing multimorbidity was consistently associated with greater length of stay (LOS) during the index ACS hospitalisation in a number of different contexts. In an Australian study of *n* = 1,488 adults aged ≥ 65 years admitted with NSTE-ACS, patients with ≥ 2 pre-existing long-term conditions had a greater mean LOS (5.8 *vs.* 4.1 days; *p* = < 0.001)[30]. Similar findings were reported for patients aged ≥ 70 admitted with MI in Sweden, for both those with NSTE-ACS (7.6 *vs*. 7.2 days; *p* < 0.001) and STEMI (7.4 *vs*. 6.9 days; *p* < 0.001)[12, 13]. Patients with pre-existing multimorbidity were also reported to have a longer mean LOS in the TRACE-CORE study (6.0 *vs.* 3.8 days; *p* < 0.001). Similar findings were reported in a series of papers describing patients admitted to hospitals participating in the Worcester Heart Attack Study[35-38].

**Unplanned readmission**

The presence of pre-existing multimorbidity was associated with unplanned hospital readmission following ACS. In patients aged ≥ 70 in SWEDEHEART, those with multimorbidity had greater rates of 30-day and 1-year all-cause readmissions. This was observed in both STEMI (30-day: 22.7 *vs* 17.6%, *p* < 0.0001; 1-year: 56.2 *vs*. 44.5%, *p* < 0.0001) and NSTE-ACS (30-day: 26.4 *vs*. 21%, *p* < 0.0001; 1-year: 63.4 *vs*. 49.1%; *p* < 0.0001)[12, 13]. Similar trends were noted at 30-days and 1-year for cause-specific readmission due to ACS, stroke or TIA, heart failure or major bleeding. A number of reports from the Worcester Heart Attack Study[35, 38], report similar findings. Conversely, in *n* = 120 patients aged 80 years or older presenting with NSTE-ACS, Nguyen *et al*. (2020) reported no association between multimorbidity status (HR 0.73, 95% CI 0.32 – 1.66; *p* = 0.455)[12, 13].

**Healthcare and hospitalisation costs**

Few studies reported the effect of pre-existing multimorbidity on healthcare costs in the context of ACS. A large US-based administrative dataset of patients admitted to hospital with ACS reported the cost of hospitalisation by multimorbidity burden (number of long-term conditions)[44]. The cost of hospitalisation increased in stepwise fashion with regard to the number of pre-existing long-term conditions: those patients with 0 comorbidities incurred median (interquartile range) hospital costs of $17,362 (14,501 – 21,632), compared to $17,630 (14,226 – 22,085), $18,188 (14,819 – 22,572), $19,384 (14,960 – 23,822), $21,193 (15,269 – 25,664) and $25,924 (16,394 – 31,146) for those with 1, 2, 3, 4 and ≥ 5 long-term conditions, respectively.

Another study reported total healthcare-associated expenditure for the year in which a patient has an ACS admission in patients with ACS only, ACS and HF, ACS and AF and ACS, AF and HF[10]. Those with a greater burden of long-term conditions had markedly greater all-cause mean healthcare expenditures: compared to those with ACS alone, those with HF, AF or both AF and HF incurred $5,073 (95% CI: 719 – 9,427), $11,297 (5,610 – 16,985) and $15,761 (4,784 – 26,738) greater costs, respectively[10].

**Patient Reported Outcome Measures**

Five studies reported the association of multimorbidity with one or more patient reported outcome measures (PROMs) in the post-MI setting[7, 9, 17, 24, 26]. The main findings, which relate to (i) quality of life, (ii) fatigue, physical activity and vital exhaustion, and (iii) life chaos are summarised below.

*Health-related quality of life (HRQoL).* Latent class analysis was applied to 9,566 patients hospitalised in the UK with acute MI (3,908 with STEMI; 5,658 with NSTEMI) to group patients into three multimorbidity classes, which indicated ‘mild’, ‘moderate’ and ‘severe’ multimorbidity[26]. Those patients with severe multimorbidity reported worse health-related quality of life (as measured using the EuroQol 5-dimension [EQ5D] and visual analogue [EQ-VAS] instruments; lower score reflects worse HRQoL) at baseline and at all follow-up points (1, 6 and 12 months). Each group reported their lowest HRQoL during their MI hospitalisation. Whereas the HRQoL for individuals in the mild multimorbidity class recovered to levels comparable to those of the UK general population at 6-months post-MI, those with a moderate-to-severe multimorbidity burden had persistently worse HRQoL to at least 12-months post-MI.

*Fatigue, physical activity and vital exhaustion*. Fatigue was measured in a convenience sample of older women (≥ 65 years) identified post-MI using the Revised Piper Fatigue Scale (higher score reflects greater fatigue). Multimorbidity was noted to negatively correlate with physical activity (*r* = -0.257; *p* < 0.05) and positively correlate with BMI (*r* = 0.234; *p* < 0.01)[7]. However, multimorbidity burden was not associated with post-MI fatigue in multivariable linear regression analysis (β = -0.057; p = 0.568). Subsequently, a cross-sectional survey of determinants of fatigue (also quantified using the Revised Piper Fatigue Scale) in *n* = 65 patients, aged 65 and older, 6 – 8 months post-MI, was reported[17]. Participants with greater multimorbidity had a higher BMI (*t* = -2.49; *p* = 0.02) and a greater fatigue score (*t* = -2.74; *p* = 0.007). Multimorbidity remained associated with fatigue in multivariable regression adjusted for age, sex and marital status. In another study, fatigue was quantified in *n* = 305 consecutive MI patients aged ≤ 80 years using the Maastricht Questionnaire for vital exhaustion (higher score reflects greater exhaustion)[24]. Greater pre-existing multimorbidity was associated with vital exhaustion in age- and sex-adjusted analysis of covariance (*p* < 0.01 for linear trend).

*Life chaos*. A cross-sectional study of *n* = 242 patients with MI in Malaysia evaluated the association of multimorbidity (≥ 2 long-term conditions) with ‘life chaos’ as measured using the Modified Confusion, Hubbub, and Order Scale (CHAOS-6; higher score reflects greater life chaos)[9]. Multimorbidity was associated with greater multimorbidity in both univariate analysis (*p* = 0.005) and multivariable linear regression analysis adjusted for age, household income, perceived financial insecurity and perceived poor health status (β = 1.7, 95% CI 0.3 – 3.0; *p* = 0.014).

**Secular trends in multimorbidity prevalence**

A number of studies have analysed the prevalence of multimorbidity in those presenting with ACS at different time points. All studies that reported serial estimates suggest that the prevalence of multimorbidity is increasing in those presenting with ACS, year-on-year. A large, retrospective analysis of US administrative data reported that the proportion of patients presenting with MI with ≥ 2 long-term conditions increased in stepwise fashion, from 56% in 2004 to 73% in 2014[44]. This is supported by a number of reports from the Worcester Heart Attack Study, which has reported over four decades, using a similar methodology and definitions of conditions at each time point. Serial measurements of the prevalence of multimorbidity included: 24.2% (1975 to 1984), 34% (1990 to 1991), 49.4% (2005 to 2007)[25] and 53.2% (2011 to 2015)[38]. A similar secular trend has also been described by a number of other studies[30, 33].

**References**

1. Alsawas, M., et al., *Gender disparities among hospitalised patients with acute myocardial infarction, acute decompensated heart failure or pneumonia: Retrospective cohort study.* BMJ Open, 2019. **9**(1): p. e022782.

2. Attar, R., et al., *Major adverse cardiovascular events following acute coronary syndrome in patients with bipolar disorder.* INTERNATIONAL JOURNAL OF CARDIOLOGY, 2022. **363**: p. 1-5.

3. Bagai, A., et al., *Multimorbidity, functional impairment, and mortality in older patients stable after prior acute myocardial infarction: Insights from the TIGRIS registry.* Clinical cardiology, 2022.

4. Bagai, A., et al., *MULTI-MORBIDITY, FUNCTIONAL IMPAIRMENT AND MORTALITY IN OLDER PATIENTS AFTER ACUTE MYOCARDIAL INFARCTION: A REPORT FROM THE TIGRIS REGISTRY.* Journal of the American College of Cardiology, 2020. **75**(11): p. 201.

5. Canivell, S., et al., *Prognosis of cardiovascular and non-cardiovascular multimorbidity after acute coronary syndrome.* PloS one, 2018. **13**(4): p. e0195174.

6. Chen, H.Y., et al., *A 35-Year Perspective (1975 to 2009). into the Long-Term Prognosis and Hospital Management of Patients Discharged from the Hospital After a First Acute Myocardial Infarction.* AMERICAN JOURNAL OF CARDIOLOGY, 2015. **116**(1): p. 24-29.

7. Crane, P.B., *Fatigue and physical activity in older women after myocardial infarction.* HEART & LUNG, 2005. **34**(1): p. 30-38.

8. Dunn, S.L., et al., *Hopelessness and its effect on cardiac rehabilitation exercise participation following hospitalization for acute coronary syndrome.* Journal of cardiopulmonary rehabilitation and prevention, 2009. **29**(1): p. 32-9.

9. Ganasegeran, K. and A. Rashid, *Factors Associated with Perceived Life Chaos among Post-Myocardial Infarction Survivors in a Malaysian Cardiac Care Facility.* Medicina (Kaunas, Lithuania), 2018. **54**(5).

10. Ghushchyan, V., K.V. Nair, and R.L. Page, *Indirect and direct costs of acute coronary syndromes with comorbid atrial fibrillation, heart failure, or both.* Vascular Health and Risk Management, 2015. **11**: p. 25-34.

11. Gouda, P., et al., *Long-term risk of death and recurrent cardiovascular events following acute coronary syndromes.* PLOS ONE, 2021. **16**(7).

12. Gudnadottir, G.S., et al., *Multimorbidity and Readmissions in Older People with Acute Coronary Syndromes.* Cardiology, 2022. **147**(2): p. 121-132.

13. Gudnadottir, G.S., et al., *Outcomes after STEMI in old multimorbid patients with complex health needs and the effect of invasive management.* American heart journal, 2019. **211**: p. 11-21.

14. Gutacker, N., K. Bloor, and R. Cookson, *Comparing the performance of the Charlson/Deyo and Elixhauser comorbidity measures across five European countries and three conditions.* European journal of public health, 2015. **25 Suppl 1**: p. 15-20.

15. Hall, M., et al., *Multimorbidity and survival for patients with acute myocardial infarction in England and Wales: Latent class analysis of a nationwide population-based cohort.* PLoS Med, 2018. **15**(3): p. e1002501.

16. Yadegarfar, M.E., et al., *Association of treatments for acute myocardial infarction and survival for seven common comorbidity states: A nationwide cohort study.* BMC Medicine, 2020. **18**(1): p. 231.

17. Horne, C.E., S. Johnson, and P.B. Crane, *Comparing comorbidity measures and fatigue post myocardial infarction.* Applied nursing research : ANR, 2019. **45**: p. 1-5.

18. Hudzik, B., et al., *Prognostic impact of multimorbidity in patients with type 2 diabetes and ST-elevation myocardial infarction.* Oncotarget, 2017. **8**(61): p. 104467-104477.

19. Hudzik, B., et al., *Association between multimorbidity and mean platelet volume in diabetic patients with acute myocardial infarction.* Acta diabetologica, 2018. **55**(2): p. 175-183.

20. Jain, S., et al., *Defining Multimorbidity in Older Patients Hospitalized with Medical Conditions.* Journal of general internal medicine, 2022.

21. Johnman, C., et al., *Clinical outcomes following radial versus femoral artery access in primary or rescue percutaneous coronary intervention in Scotland: retrospective cohort study of 4534 patients.* Heart (British Cardiac Society), 2012. **98**(7): p. 552-7.

22. King, R. and D. Giedrimiene, *Primary care physician services and the frequency of comorbidities in patients with acute myocardial infarction.* European Journal of Preventive Cardiology, 2021. **28**(SUPPL 1): p. i248.

23. King, R. and D. Giedrimiene, *The impact of comorbidities on preventive care of patients with myocardial infarction: is there still a gender gap?* European Journal of Preventive Cardiology, 2022. **29**(SUPPL 1): p. i62.

24. McGowan, L., et al., *The relationship between vital exhaustion, depression and comorbid illnesses in patients following first myocardial infarction.* JOURNAL OF PSYCHOSOMATIC RESEARCH, 2004. **57**(2): p. 183-188.

25. McManus, D.D., et al., *Multiple cardiovascular comorbidities and acute myocardial infarction: temporal trends (1990-2007) and impact on death rates at 30 days and 1 year.* Clinical epidemiology, 2012. **4**: p. 115-23.

26. Munyombwe, T., et al., *Association of multimorbidity and changes in health-related quality of life following myocardial infarction: a UK multicentre longitudinal patient-reported outcomes study.* BMC Medicine, 2021. **19**(1): p. 227.

27. Navathe, A.S., et al., *Does Admission to a Teaching Hospital Affect Acute Myocardial Infarction Survival?* ACADEMIC MEDICINE, 2013. **88**(4): p. 475-482.

28. Nguyen, H.L., et al., *Prevalence of comorbidities and their impact on hospital management and short-term outcomes in Vietnamese patients hospitalized with a first acute myocardial infarction.* PloS one, 2014. **9**(10): p. e108998.

29. Nguyen, T.V., et al., *Non-ST elevation acute coronary syndrome in patients aged 80 years or older in Vietnam: An observational study.* PloS one, 2020. **15**(6): p. e0233272.

30. Ofori-Asenso, R., et al., *Prevalence and impact of non-cardiovascular comorbidities among older adults hospitalized for non-ST segment elevation acute coronary syndrome.* Cardiovascular diagnosis and therapy, 2019. **9**(3): p. 250-261.

31. Sanchis, J., et al., *Comorbidity assessment for mortality risk stratification in elderly patients with acute coronary syndrome.* European journal of internal medicine, 2019. **62**: p. 48-53.

32. Sanchis, J., et al., *Comorbidity burden and revascularization benefit in elderly patients with acute coronary syndrome.* REVISTA ESPANOLA DE CARDIOLOGIA, 2021. **74**(9): p. 765-772.

33. Sun, J.Y., et al., *[Trends regarding the 30-day readmission rates in patients discharged for acute myocardial infarction in Beijing].* Zhonghua liu xing bing xue za zhi = Zhonghua liuxingbingxue zazhi, 2020. **41**(6): p. 866-871.

34. Tisminetzky, M., et al., *Multiple Chronic Conditions and Psychosocial Limitations in Patients Hospitalized with an Acute Coronary Syndrome.* The American journal of medicine, 2016. **129**(6): p. 608-14.

35. Tisminetzky, M., et al., *Trends in the Magnitude of, and Patient Characteristics Associated With, Multiple Hospital Readmissions After Acute Myocardial Infarction.* The American journal of cardiology, 2016. **118**(8): p. 1117-1122.

36. Tisminetzky, M., et al., *Magnitude and impact of multiple chronic conditions with advancing age in older adults hospitalized with acute myocardial infarction.* International journal of cardiology, 2018. **272**: p. 341-345.

37. Tisminetzky, M., et al., *Impact of cardiac- and noncardiac-related conditions on adverse outcomes in patients hospitalized with acute myocardial infarction.* Journal of comorbidity, 2019. **9**: p. 2235042X19852499.

38. Tisminetzky, M., et al., *Temporal Trends and Patient Characteristics Associated with 30-Day Hospital Readmission Rates after a First Acute Myocardial Infarction.* AMERICAN JOURNAL OF MEDICINE, 2021. **134**(9): p. 1127-1134.

39. Turner, R.M., et al., *Multimorbidity, polypharmacy, and drug-drug-gene interactions following a non-ST elevation acute coronary syndrome: analysis of a multicentre observational study.* BMC medicine, 2020. **18**(1): p. 367.

40. Worrall-Carter, L., et al., *Impact of comorbidities and gender on the use of coronary interventions in patients with high-risk non-ST-segment elevation acute coronary syndrome.* Catheterization and cardiovascular interventions : official journal of the Society for Cardiac Angiography & Interventions, 2016. **87**(4): p. E128-36.

41. Worrall-Carter, L., et al., *Gender Differences in Presentation, Coronary Intervention, and Outcomes of 28,985 Acute Coronary Syndrome Patients in Victoria, Australia.* Women's health issues : official publication of the Jacobs Institute of Women's Health, 2016. **26**(1): p. 14-20.

42. Yan, Y., et al., *[Multimorbidity in elderly patients with acute coronary syndrome: insights from BleeMACS registry].* Zhonghua Xin Xue Guan Bing Za Zhi, 2022. **50**(5): p. 443-449.

43. Yang, X.D., et al., *Medical comorbidities at admission is predictive for 30-day in-hospital mortality in patients with acute myocardial infarction: analysis of 5161 cases.* JOURNAL OF GERIATRIC CARDIOLOGY, 2011. **8**(1): p. 31-34.

44. Zhang, F., et al., *Temporal Trends in Comorbidity Burden and Impact on Prognosis in Patients With Acute Coronary Syndrome Using the Elixhauser Comorbidity Index Score.* The American journal of cardiology, 2020. **125**(11): p. 1603-1611.

45. Zykov, M.V., et al., *[Combined use of the GRACE ACS risk score and comorbidity indices to increase the effectiveness of hospital mortality risk assessment in patients with acute coronary syndrome].* Terapevticheskii arkhiv, 2022. **94**(7): p. 816-821.

46. Munn, Z., et al., *Methodological guidance for systematic reviews of observational epidemiological studies reporting prevalence and cumulative incidence data.* Int J Evid Based Healthc, 2015. **13**(3): p. 147-53.

47. Munn, Z., et al., *The development of a critical appraisal tool for use in systematic reviews addressing questions of prevalence.* Int J Health Policy Manag, 2014. **3**(3): p. 123-8.

48. ROBINS-E Development Group, et al., *Risk Of Bias In Non-randomized Studies - of Exposure (ROBINS-E)  Checklist. Launch Version*. 2022.
